# Single-Cell Transcriptomics Uncovers Zonation of Function in the Mesenchyme during Liver Fibrosis

**DOI:** 10.1016/j.celrep.2019.10.024

**Published:** 2019-11-12

**Authors:** Ross Dobie, John R. Wilson-Kanamori, Beth E.P. Henderson, James R. Smith, Kylie P. Matchett, Jordan R. Portman, Karolina Wallenborg, Simone Picelli, Anna Zagorska, Swetha V. Pendem, Thomas E. Hudson, Minnie M. Wu, Grant R. Budas, David G. Breckenridge, Ewen M. Harrison, Damian J. Mole, Stephen J. Wigmore, Prakash Ramachandran, Chris P. Ponting, Sarah A. Teichmann, John C. Marioni, Neil C. Henderson

**Affiliations:** 1 Centre for Inflammation Research, The Queen’s Medical Research Institute, Edinburgh BioQuarter, University of Edinburgh, Edinburgh EH16 4TJ, UK; 2 Karolinska Institutet (KI), Science for Life Laboratory, Tomtebodavägen 23, Solna 171 65, Sweden; 3 Gilead Sciences, Foster City, CA 94404, USA; 4 Clinical Surgery, University of Edinburgh, Royal Infirmary of Edinburgh, Edinburgh EH16 4SA, UK; 5 MRC Human Genetics Unit, MRC Institute of Genetics and Molecular Medicine at the University of Edinburgh, Edinburgh EH4 2XU, UK; 6 Wellcome Trust Sanger Institute, Wellcome Genome Campus, Hinxton, Cambridge CB10 1SA, UK; 7 European Molecular Biology Laboratory, European Bioinformatics Institute (EMBL-EBI), Hinxton, Cambridge CB10 1SD, UK; 8 Theory of Condensed Matter Group, The Cavendish Laboratory, University of Cambridge, Cambridge CB3 0HE, UK; 9 Cancer Research UK Cambridge Institute, University of Cambridge, Li Ka Shing Centre, Cambridge CB2 0RE, UK

**Keywords:** liver fibrosis, mesenchyme, hepatic stellate cells, single-cell RNA sequencing, zonation

## Abstract

Iterative liver injury results in progressive fibrosis disrupting hepatic architecture, regeneration potential, and liver function. Hepatic stellate cells (HSCs) are a major source of pathological matrix during fibrosis and are thought to be a functionally homogeneous population. Here, we use single-cell RNA sequencing to deconvolve the hepatic mesenchyme in healthy and fibrotic mouse liver, revealing spatial zonation of HSCs across the hepatic lobule. Furthermore, we show that HSCs partition into topographically diametric lobule regions, designated portal vein-associated HSCs (PaHSCs) and central vein-associated HSCs (CaHSCs). Importantly we uncover functional zonation, identifying CaHSCs as the dominant pathogenic collagen-producing cells in a mouse model of centrilobular fibrosis. Finally, we identify LPAR1 as a therapeutic target on collagen-producing CaHSCs, demonstrating that blockade of LPAR1 inhibits liver fibrosis in a rodent NASH model. Taken together, our work illustrates the power of single-cell transcriptomics to resolve the key collagen-producing cells driving liver fibrosis with high precision.

## Highlights

• scRNA-seq reveals spatial zonation of hepatic stellate cells (HSCs)
• HSCs partition into topographically diametric lobule regions
• Functional zonation of HSCs during centrilobular injury-induced fibrosis is uncovered
• LPAR1 is a therapeutic target on pathological central vein-associated HSC

## Graphical Abstract

**Figure:**
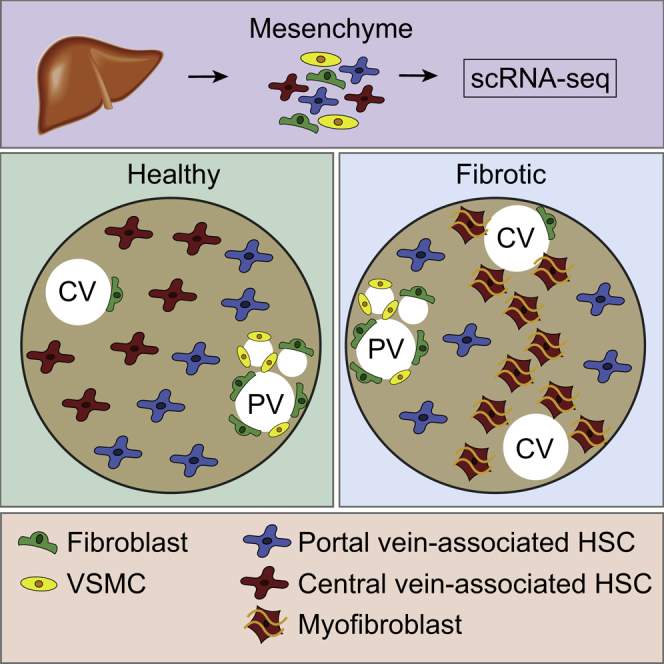

## Teaser

Dobie et al. use scRNA-seq to reveal spatial and functional zonation of hepatic stellate cells (HSCs) across the hepatic lobule, identifying central vein-associated HSCs as the dominant pathogenic collagen-producing cells during centrilobular injury-induced fibrosis. This illustrates the power of scRNA-seq to resolve the key collagen-producing cells driving liver fibrosis.

## Introduction

Liver cirrhosis is a major global healthcare burden, with an estimated 844 million people suffering from chronic liver disease worldwide (Marcellin and Kutala, 2018). Mortality rates secondary to liver cirrhosis continue to increase, with no Food and Drug Administration (FDA)- or European Medicines Agency (EMA)-approved antifibrotic treatments currently available, and liver transplantation only accessible to a select few (Friedman et al., 2018, Koyama et al., 2016, Tapper and Parikh, 2018). An ideal antifibrotic therapy would specifically target the pathogenic collagen-producing cell population without perturbing homeostatic mesenchymal function. Therefore, increasing our understanding of the precise cellular and molecular mechanisms regulating liver fibrosis is fundamental to the rational design and development of effective, highly targeted anti-fibrotic therapies for patients with chronic liver disease (Ramachandran and Henderson, 2016, Trautwein et al., 2015).

Myofibroblasts are the key source of pathogenic extracellular matrix deposition during hepatic fibrogenesis and therefore have attracted considerable interest as a potential therapeutic target (Dobie and Henderson, 2016, Friedman, 2015, Hinz et al., 2012, Kisseleva, 2017). Although different mesenchymal cell types have been proposed as the predominant source of myofibroblasts following liver injury (Iwaisako et al., 2014, Kisseleva et al., 2006, Li et al., 2013, Mederacke et al., 2013), recent studies suggest that hepatic stellate cells (HSCs), first described by Kupffer in 1876 as vitamin A^+^ lipid droplet-containing cells that reside in the perisinusoidal space of the liver (Wake, 1971), are the dominant contributors to the myofibroblast pool independent of the etiology of liver fibrosis (Iwaisako et al., 2014, Mederacke et al., 2013). Furthermore, since the discovery 35 years ago that HSCs are major collagen-producing cells in the liver (Friedman et al., 1985, de Leeuw et al., 1984), these cells have been regarded as a functionally homogeneous population, with the potential to transition to the activated, collagen-secreting myofibroblast phenotype thought to be equally distributed across all HSCs.

Single-cell RNA sequencing (scRNA-seq) is transforming our understanding of disease pathogenesis (Lee et al., 2017, Stubbington et al., 2017, Zepp et al., 2017). Here, we use scRNA-seq to resolve the hepatic mesenchyme in an unbiased manner at high resolution, analyzing the transcriptomes of over 30,000 hepatic mesenchymal cells. Our data: (1) deconvolve the hepatic mesenchyme in healthy and fibrotic mouse liver; (2) reveal spatial zonation of HSCs across the hepatic lobule; (3) generate gene signatures and markers that partition HSCs into two topographically diametric lobule regions, namely portal vein-associated HSCs (PaHSCs) and central vein-associated HSCs (CaHSCs); (4) importantly, uncover functional zonation of HSCs, identifying that CaHSCs, but not PaHSCs, are the dominant pathogenic collagen-producing cells in a mouse model of centrilobular liver injury; and (5) identify LPAR1 as a therapeutic target on collagen-producing HSCs and demonstrate that pharmacological antagonism of LPAR1 inhibits liver fibrosis. These studies allow us to further define and resolve the spatial, cellular, and molecular complexity present within the hepatic fibrotic niche. Our work highlights the power of scRNA-seq in identifying the key collagen-producing cells driving centrilobular liver fibrosis with high precision and therefore should serve as a framework for the high-resolution identification of the critical pathogenic cells and related therapeutic targets in a broad range of fibrotic diseases.

## Results

### Deconvolution of the Mouse Hepatic Mesenchyme Identifies Three Distinct Subpopulations in Liver Homeostasis

**Figure 1:**
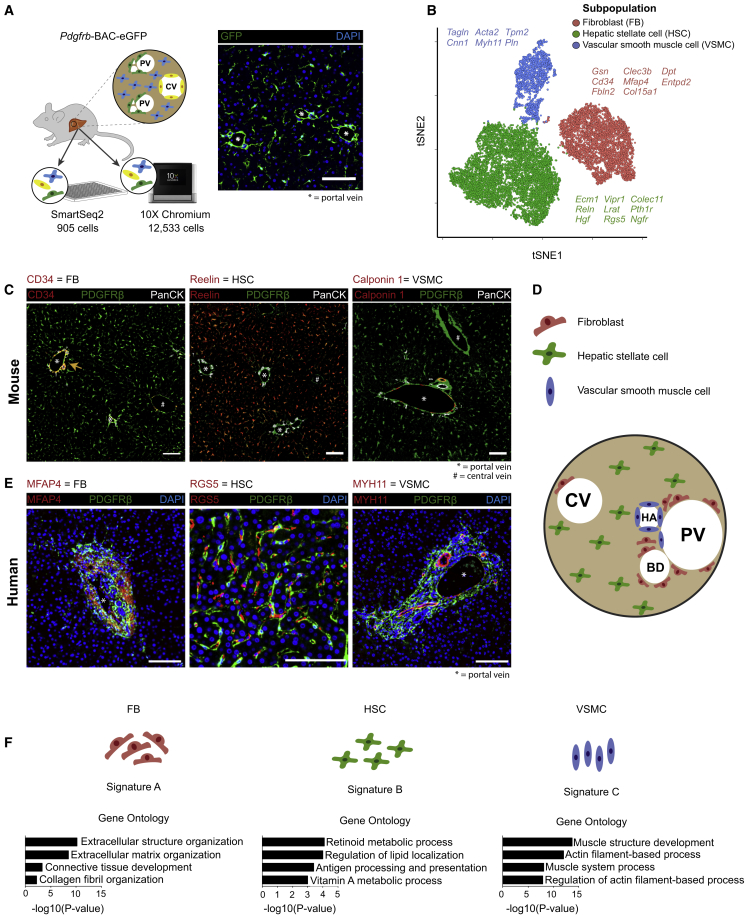
Deconvolution of the Mouse Hepatic Mesenchyme Identifies Three Distinct Subpopulations in Liver Homeostasis (A) Overview: representative immunofluorescence image depicts GFP reporting in the liver of healthy *Pdgfrb*-BAC-eGFP reporter mice. Scale bar, 100 μm; portal vein (^∗^) as indicated. CV, central vein; PV, portal vein. GFP^+^ cells were processed for droplet- and plate-based scRNA-seq. (B) t-Distributed stochastic neighbor embedding (t-SNE) visualization: 12,533 mesenchymal cells (median nGene = 2,268, nUMI = 5,725) cluster into three subpopulations. Selected marker genes are listed alongside each cluster. (C) Representative immunofluorescence images of healthy murine livers: CD34/Reelin/Calponin 1 (red), PDGFRβ (green), PanCK (white). Scale bar, 100 μm; portal vein (^∗^) and central vein (#) as indicated. Yellow arrow indicates CD34^+^ fibroblasts. (D) Schematic representation of the topography of the three identified mesenchymal subpopulations in the liver. CV, central vein; PV, portal vein; HA, hepatic artery; BD, bile duct. (E) Representative immunofluorescence images of healthy human livers: MFAP4/RGS5/MYH11 (red), PDGFRβ (green), DAPI (blue). Scale bar, 100 μm; portal vein (^∗^) as indicated. (F) GO enrichment terms associated with signatures A–C corresponding to the three identified mesenchymal subpopulations. See also Figures S1, S2, and S3.

We used a *Pdgfrb*-GFP knockin reporter mouse to label all mesenchymal cells in the mouse liver (Figure 1A). This reporter strain has previously been shown to label all mesenchymal cells (including HSCs) (Henderson et al., 2013). Here, we show that the *Pdgfrb*-GFP mouse labeled PDGFRβ^+^ cells in liver with high efficiency and specificity (Figure S1A). Two independent digestion protocols and gating strategies were used to isolate the different GFP^+^ mesenchymal cell populations (Figure S1B; STAR Methods). To initially characterize the hepatic mesenchyme at single-cell resolution, we used the 10X Chromium protocol to sequence 12,533 cells from mice (n = 3 digestion protocol 1; n = 3 digestion protocol 2) at a mean read depth of ∼85 K reads per cell, which show negligible endothelial, epithelial, and leucocyte contamination (Figures 1A, S1C, and S1D).

We identified three subpopulations of mesenchymal (PDGFRβ^+^) cells (Figure 1B) with distinct sets of marker genes (Figure S2A; Table S1). Identifying highly specific marker genes (Figure S2B) and performing immunofluorescence co-staining (Figure 1C) validated the three mesenchymal subpopulations and delineated their topography. We found that CD34^+^ PDGFRβ^+^ cells reside primarily in the portal niche, adjacent to PanCK^+^ biliary epithelial cells, with rare cells also found around the central vein, possibly representing second layer cells (Figure S2C) (Bhunchet and Wake, 1992). Reelin^+^ PDGFRβ^+^ cells were found in the perisinusoidal space throughout the parenchyma (Figure S2D). Calponin 1^+^ PDGFRβ^+^ cells were located within both the hepatic artery and the portal vein walls (Figure S2E). Given the topographic distribution of these three mesenchymal subpopulations, we labeled them as fibroblasts (FBs), HSCs, and vascular smooth muscle cells (VSMCs), respectively (Figure 1D). We annotated the CD34^+^ mesenchymal subpopulation as FBs, which are known to be major mediators of matrix turnover in the portal niche (Wells, 2014). The Reelin^+^ subpopulation represents HSCs, located throughout the parenchyma, with functions including vitamin A storage and antigen presentation (Friedman, 2008, Winau et al., 2007). Finally, the topography of the Calponin 1^+^ subpopulation is consistent with a VSMC phenotype (Patel et al., 2016).

To determine whether similar mesenchymal subpopulations exist in healthy human liver, we performed immunofluorescence co-staining using genes identified as markers in the mouse dataset (Figure 1E). Akin to our findings in mouse liver, we identified three topographically distinct mesenchymal subpopulations. MFAP4^+^ PDGFRβ^+^ cells were confined to the portal niche, consistent with the CD34^+^ PDGFRβ^+^ subpopulation observed in the portal niche of mouse liver. RGS5^+^ PDGFRβ^+^ cells were found in locations throughout the hepatic parenchyma, consistent with HSCs. MYH11^+^ PDGFRβ^+^ cells were located around portal vein walls, consistent with VSMCs.

To assess the functional profile of the three mouse mesenchymal subpopulations, we generated self-organizing maps using the *SCRAT* R package (Camp et al., 2017) to visualize coordinately expressed gene groups across the transcriptomic landscape (Figure S2F). We identified three metagene signatures, denoted as A–C, that strongly define the subpopulations (Table S2). Signature A, enriched for gene ontology (GO) terms relating to extracellular structure organization, defined both FBs and VSMCs mesenchymal subpopulations. Signature B defined the HSCs subpopulation and was enriched for terms including retinoid metabolic process and antigen processing and presentation. Signature C defined VSMCs exclusively and was enriched for terms such as actin filament-based processes (Figures 1F and S2F).

Using a single-cell approach also allowed us to interrogate “traditional” hepatic mesenchymal markers at high resolution. We found that certain historic HSC markers, such as *Des* and *Vim*, were expressed variably across all three mesenchymal subpopulations (Figure S2G). In keeping with recent findings (Mederacke et al., 2013), *Gfap* expression was negligible in our dataset. We confirmed *Lrat* and *Reln* as specific markers for HSCs within the hepatic mesenchyme (Lua et al., 2016, Mederacke et al., 2013), and *Ngfr* displayed a spectrum of expression across the HSC population. *Pdgfra* expression was confined to the FB and HSC subpopulations as opposed to *Pdgfrb*, which was pan-mesenchymal.

To reproduce our 10X Chromium-based findings, and to assess whether a plate-based full-length transcript approach would identify similar mesenchymal subpopulations, we also obtained scRNA-seq data of PDGFRβ^+^ cells in liver using SmartSeq2 (SSeq2). We sequenced 905 cells isolated using both digestion protocols at a mean read depth of ∼456 K reads per cell. Analysis of this SSeq2 dataset identified the same three mesenchymal subpopulations (Figure S3A; Table S1) with negligible non-mesenchymal cell contamination (Figure S3B). This alternative sequencing approach replicated our findings both in terms of the marker genes identified previously and the GO profiles generated using SCRAT (Figures S3C–S3G; Table S2).

### Uncovering HSC Zonation across the Healthy Liver Lobule

**Figure 2:**
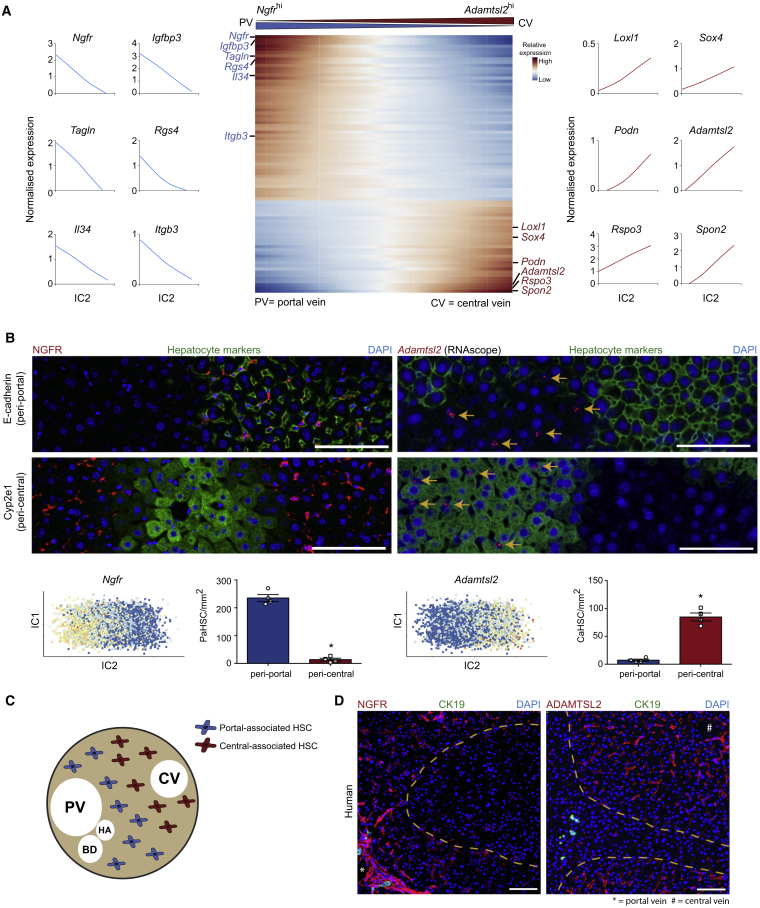
Uncovering HSC Zonation across the Healthy Liver Lobule (A) Heatmap of relative expression (center): cubic smoothing spline curves fitted to markers of HSC zonation and plotted along IC2; genes are thresholded and ordered on their contribution to IC2, with top-most genes displaying the strongest negative correlation with bottom-most genes. Cells columns, genes rows. Zonation profiles for exemplar genes shown left and right. (B) Representative immunofluorescence and RNAscope images of healthy murine livers: NGFR/*Adamtsl2* (RNAscope) (red), E-cadherin/Cyp2e1 (green), DAPI (blue). Scale bar, 100 μm. Yellow arrows indicate *Adamtsl2*^+^ cells. ICA visualizations (below): *Ngfr* and *Adamtsl2* expression on the first and second independent components of the HSC subpopulation in homeostatic murine liver. Bar plots (below): number of PaHSCs (left; n = 4) and CaHSCs (right; n = 4) per mm^2^ in peri-portal and peri-central regions; error bars SEM, Mann-Whitney test, ^∗^p < 0.05. (C) Schematic representation of the topography of the two HSC subpopulations in healthy liver lobule. CV, central vein; PV, portal vein; HA, hepatic artery; BD, bile duct. (D) Representative immunofluorescence images of healthy human livers: NGFR/ADAMTSL2 (red), CK19 (biliary epithelial cell marker; green), DAPI (blue). Scale bar, 100 μm; portal vein (^∗^) and central vein (#) as indicated. Yellow dashed lines mark areas of low/neg marker staining. See also Figures S4 and S5.

The micro-architecture of the hepatic lobule displays highly ordered three-dimensional structural motifs consisting of a portal triad, hepatocytes arranged in linear cords between a sinusoidal capillary network, and a central vein and is highly conserved across species (Burke et al., 2009, Gebhardt, 1992, Kietzmann, 2017). Given the known zonation of hepatocytes (Halpern et al., 2017, Lamers et al., 1989) and endothelia (Halpern et al., 2018) across the liver lobule, and having observed variable patterns of gene expression in the HSC population (Figure S2G), we investigated the existence of similar zonation in HSCs. We used independent component analysis (ICA) to identify a set of highly variable genes in uninjured HSCs (Figure 2A). Thresholding on the gene weight loadings along this component, we extracted 81 genes consisting of two opposed signatures: 52 genes associated with and including *Ngfr* and *Itgb3* and 29 genes associated with and including *Adamtsl2* and *Rspo3* (Figure 2A; Table S1). Supervised clustering using this signature allowed us to separate the HSCs into two further subpopulations.

To determine the topography of these subpopulations, we selected marker genes using the following criteria: (1) high gene weight loading on the independent component (IC) (Figure 2A), (2) mesenchyme specificity (Figure S4A), and (3) within the mesenchyme, greatest specificity to HSCs (Figure S4B). We identified *Adamtsl2* and *Ngfr* as the best candidate markers. Using a combination of a highly sensitive modified *in situ* RNA hybridization procedure (RNAscope) and immunofluorescence staining, we confirmed *Adamtsl2* and NGFR to be mesenchymal markers each labeling a subpopulation of HSCs (Figure S4A). To explore how this topography related to zonally distributed hepatocytes, we co-stained with peri-portal (E-cadherin) and peri-central (Cyp2e1) hepatocyte markers (Doi et al., 2007, Rocha et al., 2015). We found NGFR^hi^ HSCs located in the same region of the liver lobule as portal vein-associated (E-cadherin^+^) hepatocytes, whereas *Adamtsl2*^hi^ HSC was located in the same region of the liver lobule as central vein-associated hepatocytes (Cyp2e1^+^) (Figures 2B and S4C). This allowed us to annotate these two HSC subpopulations as NGFR^hi^ portal vein-associated HSCs (PaHSCs) and *Adamtsl2*^hi^ central vein-associated HSCs (CaHSCs) (Figure 2C).

To determine whether healthy human liver exhibits similar HSC zonation, we performed immunofluorescence co-staining using marker genes orthologous to those that delineated mouse HSC zonation—*Ngfr* and *Adamtsl2*—with the previously identified HSC marker RGS5 (Figure S4D). Akin to our findings in mouse liver, we observed zonal expression of NGFR and ADAMTSL2 across the human liver lobule (Figure 2D).

Certain genes within our zonation signature, such as *Itgb3* and *Rspo3*, have previously been identified as landmark genes used to zonally define endothelial cells across the liver lobule (Halpern et al., 2018). Spatial mapping of these populations using RNAscope and immunofluorescence staining confirmed the presence of zonally distributed ITGB3^+^ (Integrin β3) and *Rspo3*^*+*^ HSCs within the parenchyma (Figure S5A). In line with previous studies, Integrin β3^hi^ HSCs were observed in the peri-portal region and *Rspo3*^hi^ HSCs in the peri-central region, suggesting possible spatial correlation between the endothelial and mesenchymal lineages (Figures S5A and S5B). Immunofluorescence staining of PaHSC marker NGFR and previously identified central-associated endothelial cell marker thrombomodulin (Halpern et al., 2018) further demonstrated zonation, with each marker defining a distinct region within the hepatic lobule (Figure S5C).

### HSC Populate the Fibrotic Niche in a Mouse Model of Centrilobular Fibrotic Liver Injury

Chronic carbon tetrachloride (CCl_4_) administration is a broadly utilized, highly reproducible, and robust mouse model of centrilobular liver fibrosis that recapitulates many of the features of human fibrotic liver disease (Figures S6A and S6B). To investigate mesenchymal cell heterogeneity in fibrotic mouse liver, we sequenced 10,758 *Pdgfrb*-GFP^+^ reporter cells from murine liver following 6 weeks CCl_4_ administration using the 10X Chromium workflow and performed unsupervised clustering alongside our homeostatic hepatic mesenchyme dataset (Figures S6C and S6D).

**Figure 3:**
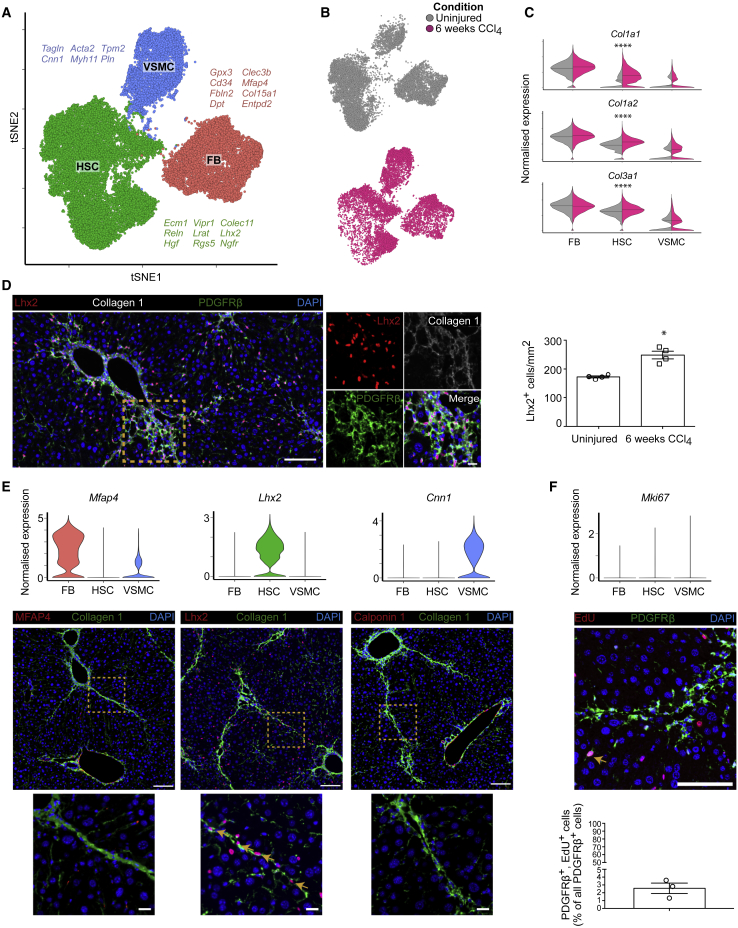
HSCs Populate the Fibrotic Niche in a Mouse Model of Centrilobular Fibrotic Liver Injury (A) t-SNE visualization: 23,291 mesenchymal cells (median nGene = 2,339, nUMI = 6,081) from uninjured and fibrotic (6 weeks CCl_4_) mouse livers cluster into three subpopulations. FB, fibroblasts; HSC, hepatic stellate cells; VSMC, vascular smooth muscle cells. Selected marker genes listed alongside each cluster. (B) t-SNE visualizations: cells from uninjured (gray) and fibrotic (pink) livers. (C) Violin plots: expression of fibrillar collagen genes (*Col1a1*, *Col1a2*, and *Col3a1*) across the three subpopulations in uninjured (gray) versus fibrotic (pink) livers, bar indicates median. Mann-Whitney test, ^∗∗∗∗^p value < 0.0001. (D) Representative immunofluorescence images of fibrotic murine liver and quantification of Lhx2^+^ HSC in fibrotic versus uninjured murine liver: Lhx2 (red), collagen 1 (white), PDGFRβ (green), DAPI (blue). Scale bar, 100 μm. Yellow dashed line marks magnified area (scale bar, 20 μm). Bar plot (right): number of Lhx2^+^ cells per mm^2^ in uninjured (n = 4) and fibrotic (n = 4) liver; error bars SEM, Mann-Whitney test, ^∗^p value < 0.05. (E) Violin plots (top): expression of mesenchymal cell subpopulation markers. Representative immunofluorescence images of fibrotic murine liver (below): MFAP4/Lhx2/Calponin 1 (red), collagen 1 (green), DAPI (blue). Scale bar, 100 μm. Yellow dashed line marks magnified area (scale bar, 20 μm). Yellow arrows indicate Lhx2^+^ cells within the fibrotic niche. (F) Violin plot (top): expression of proliferation marker *Mki67* across the three mesenchymal subpopulations. Representative immunofluorescence images of fibrotic murine liver (middle): EdU (red), PDGFRβ (green), DAPI (blue). Scale bar, 100 μm. Bar plot (bottom): percentage EdU^+^ mesenchymal cells (n = 3); error bars SEM. Yellow arrow indicates proliferating PDGFRβ^+^ cell. See also Figure S6.

We observed the same three mesenchymal subpopulations in both healthy and fibrotic livers (Figures 3A and 3B), with previously identified markers maintaining their specificity following chronic liver injury (Table S1). Expression of fibrillar collagens (*Col1a1*, *Col1a2*, and *Col3a1*) remained highest in FBs, however, a marked increase in expression of *Col1a1* was observed in HSCs following induction of fibrotic injury (Figure 3C). Although overall marker gene expression profiles remained constant between uninjured and fibrotic HSCs compared to other mesenchymal populations, we also observed decreased expression of certain marker genes including *Reln* in HSC overexpressing fibrillar collagen (Figure S6E). Immunofluorescence staining confirmed diminished Reelin positivity within the fibrotic niche (Figure S6F). In contrast, staining for Lhx2 (a mesenchyme-specific marker of HSCs in both uninjured and 6 weeks CCl_4_ liver) confirmed an expansion of HSCs within the fibrotic niche (Figures 3D, S6F, and S6G). Previous studies have shown that HSCs are the major source of pathogenic collagen-producing cells following liver injury (Iwaisako et al., 2014, Mederacke et al., 2013); in accordance we did not identify FBs and VSMCs within the fibrotic niche, as evidenced by immunofluorescence staining for markers MFAP4 and Calponin 1 (Figure 3E). We observed minimal proliferation across all three populations (2.4% of mesenchymal cells in the dataset expressed proliferation marker *Mki67*), confirmed by ethynyldeoxyuridine (EdU) staining in CCl_4_-treated livers (2.6% of mesenchymal cells; Figure 3F).

### CaHSCs Are the Dominant Pathogenic Collagen-Producing Cells in a Mouse Model of Centrilobular Fibrotic Liver Injury

**Figure 4:**
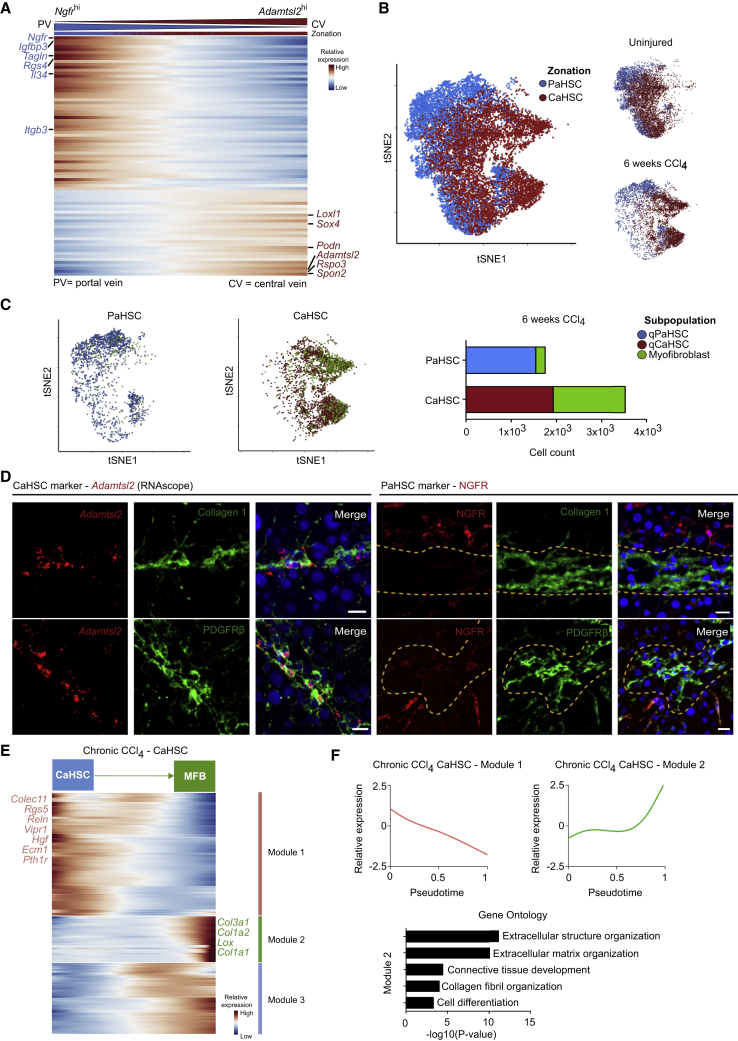
CaHSCs Are the Dominant Pathogenic Collagen-Producing Cells in a Mouse Model of Centrilobular Fibrotic Liver Injury (A) Heatmap of relative expression: cubic smoothing spline curves fitted to previously defined markers of zonation in murine HSCs, ordered by expression of *Ngfr*-associated (portal vein-associated) signature and annotated by cell condition. Cells columns, genes rows. (B) t-SNE visualizations: clustering HSCs from uninjured and fibrotic (6 weeks CCl_4_) livers on zonation signature separates them into distinct PaHSC and CaHSC clusters. (C) t-SNE visualizations: thresholding HSCs on expression of fibrillar collagen genes (*Col1a1*, *Col1a2*, and *Col3a1*), PaHSCs (left) versus CaHSCs (middle). qPaHSCs (blue) and qCaHSCs (red), HSCs below fibrillar collagen threshold, where q = quiescent HSC state; myofibroblast (green), HSCs above fibrillar collagen threshold. Bar plot (right): cell counts for PaHSCs versus CaHSCs from fibrotic livers. Green portion of bars represent HSCs above fibrillar collagen threshold. (D) Representative immunofluorescence and RNAscope images of fibrotic livers: *Adamtsl2* (RNAscope)/NGFR (red), collagen 1/PDGFRβ (green), DAPI (blue). Scale bar, 20 μm. Yellow dashed line marks area of NGFR^lo/neg^ HSCs. (E) Heatmap of relative expression: cubic smoothing spline curves fitted to genes differentially expressed across transition from quiescent CaHSC to myofibroblast, grouped by hierarchical clustering (k = 3). Gene co-expression modules labeled right. MFB, myofibroblast. (F) Cubic smoothing spline curves fitted to averaged relative expression of all genes in module 1 and module 2 along transition from quiescent CaHSC to myofibroblast; selected GO enrichment terms for module 2 (bottom). See also Figure S7.

Since the discovery 35 years ago that HSCs are major collagen-producing cells in the liver (Friedman et al., 1985, de Leeuw et al., 1984), they have been regarded as a functionally homogeneous population, with the potential to transition to the activated, collagen-secreting myofibroblast phenotype thought to be equally distributed across all HSCs. Having identified HSC zonation in the homeostatic liver, we therefore investigated the relative contributions of PaHSCs and CaHSCs to the fibrotic process. We performed supervised clustering on the combined healthy and fibrotic HSC populations based on their expression of the 81 zonation genes previously identified in homeostatic HSCs (Table S1) and found clear conservation of the zonation genes observed in homeostasis: 51/52 PaHSC-associated genes including *Ngfr and Itgb3* and 26/29 CaHSC-associated genes including *Adamtsl2* and *Rspo3* continued to define the zonation profile of the combined HSCs cluster (Figure 4A). This again allowed delineation of HSCs into PaHSC and CaHSC subpopulations (Figure 4B).

To investigate whether our zonation signature correlated with pathogenic collagen-producing HSCs, we created a myofibroblast signature by thresholding HSCs on fibrillar collagen (*Col1a1*, *Col1a2*, and *Col3a1*) overexpression, a hallmark of HSC activation. This signature was then mapped onto our zonation profile to identify the contribution of PaHSCs and CaHSCs to the pathogenic myofibroblasts. In this way, we identify CaHSCs as the dominant pathogenic collagen-producing HSCs, representing 88% of myofibroblasts classified in this manner (Figure 4C). These findings were validated by spatial mapping of CaHSCs and PaHSCs in fibrotic liver using RNAscope and immunofluorescence staining. *Adamtsl2*^hi^ CaHSCs were located throughout the fibrotic niche whereas NGFR^hi^ PaHSCs resided predominately in the parenchyma distal to the fibrotic region. (Figures 4D and S7A). A similar difference in topography was observed with other zonation markers *Rspo3* and Integrin β3 (Figure S7B).

To further investigate the contribution of CaHSCs to pathogenic collagen production during fibrosis, we used self-organizing maps to identify metagene signatures enriched in the collagen-producing HSCs (Figure S7C; Table S2). Signature A was expressed across both PaHSCs and CaHSCs, with enriched GO terms such as retinoid metabolic process that also defined uninjured HSCs (Figure 1F). Signature B was highly enriched for terms related to collagen production, and all HSCs that expressed signature B were CaHSCs.

Having identified CaHSCs as the predominant pathogenic collagen-producing HSCs, we used the *monocle* R package to further investigate changes in gene expression within the CaHSCs. This highlighted the transition from a quiescent to a collagen-producing phenotype, with upregulation following injury of pro-fibrogenic genes including *Col1a1*, *Col1a2*, *Col3a1*, and *Lox*, with associated enriched GO terms such as extracellular structure organization and collagen fibril organization, and downregulation of uninjured HSC-related genes including *Ecm1*, *Reln*, *Hgf*, and *Rgs5* (Figures 4E and 4F; Table S3).

### CaHSCs Are the Dominant Pathogenic Collagen-Producing Cells following Acute Centrilobular Liver Injury

Acute CCl_4_-induced liver injury is characterized by significant HSC proliferation (14.4% of Lhx2^+^ HSCs) and activation to a collagen-producing myofibroblast phenotype in the centrilobular region (Figures S8A–S8C). To further interrogate the dynamics of PaHSC and CaHSC differentiation into pathogenic collagen-producing cells, we sequenced 7,260 HSCs from *Pdgfrb*-GFP reporter mice following acute CCl_4_-induced liver injury (Figures S8D and S8E).

**Figure 5:**
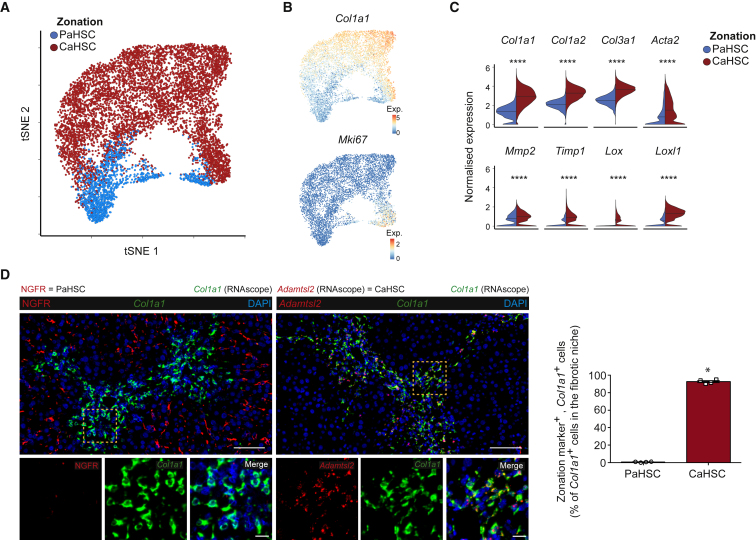
CaHSCs Are the Dominant Pathogenic Collagen-Producing Cells following Acute Centrilobular Liver Injury (A) t-SNE visualization: clustering 7,260 HSC following acute CCl_4_ administration on zonation signature separates them into distinct PaHSC and CaHSC clusters (median nGene = 3,235, nUMI = 11,373). (B) t-SNE visualizations: *Col1a1* and *Mki67* gene expression. (C) Violin plots: expression of profibrogenic genes across PaHSC and CaHSC subpopulations, bar indicates median. Mann-Whitney test, ^∗∗∗∗^p < 0.0001. (D) Representative immunofluorescence and RNAscope images of murine livers following acute CCl_4_ administration: NGFR/*Adamtsl2* (RNAscope) (red), *Col1a1* (RNAscope) (green), DAPI (blue). Scale bar, 100 μm. Yellow dashed line marks magnified area (scale bar, 20 μm). Bar plot (right): PaHSC and CaHSC *Col1a1* specificity within the fibrotic niche (n = 4); error bars SEM. Mann-Whitney test, ^∗^p < 0.05. See also Figure S8.

We used the 81 zonation genes identified in homeostatic HSCs to classify these cells into PaHSC and CaHSC subpopulations (Figures 5A and S8F). Both subpopulations contained cells expressing the known proliferation marker *Mki67*, however, only CaHSCs showed elevated levels of known genes associated with fibrogenesis (Figures 5B and 5C). Spatial mapping of these subpopulations using RNAscope combined with immunofluorescence staining confirmed the pathogenic collagen-producing cells in the fibrotic niche as overwhelmingly *Adamtsl2*^hi^ CaHSCs (Figures 5D and S8G).

### CaHSCs, but Not PaHSCs, Differentiate into Pathogenic Collagen-Producing Cells following Acute Centrilobular Liver Injury

**Figure 6:**
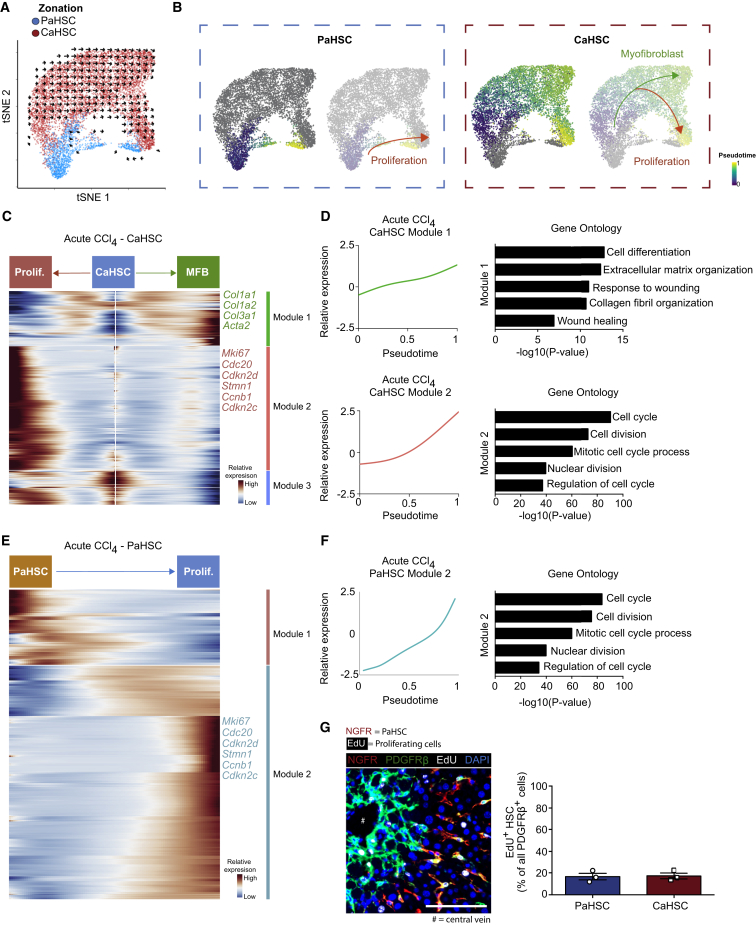
CaHSCs, but Not PaHSCs, Differentiate into Pathogenic Collagen-Producing Cells following Acute Centrilobular Liver Injury (A) t-SNE visualization: RNA velocity field (black vectors) visualized using Gaussian smoothing on regular grid, superimposed on PaHSC and CaHSC clusters. (B) Annotating pseudotemporal dynamics (purple to yellow) on PaHSC (left) and CaHSC (right) clusters. Arrows indicate simplified overall trajectory. (C) Heatmap of relative expression: cubic smoothing spline curves fitted to genes differentially expressed across quiescent CaHSC to myofibroblast (MFB) (right arrow) and across quiescent to proliferating CaHSC (left arrow) pseudotemporal trajectories, grouped by hierarchical clustering (k = 3). Gene co-expression modules labeled right. (D) Cubic smoothing spline curves fitted to averaged expression of all genes in module 1 (top) along quiescent CaHSC to myofibroblast pseudotemporal trajectory, selected GO enrichment terms (right), and module 2 (bottom) along the quiescent to proliferating CaHSC pseudotemporal trajectory, selected GO enrichment terms (right). (E) Heatmap of relative expression: cubic smoothing spline curves fitted to genes differentially expressed across quiescent to proliferating PaHSC pseudotemporal trajectory, grouped by hierarchical clustering (k = 2). Gene co-expression modules labeled right. (F) Cubic smoothing spline curves fitted to averaged expression of all genes in module 2 along quiescent to proliferating PaHSC pseudotemporal trajectory, selected GO enrichment terms (right). (G) Representative immunofluorescence image of murine liver following acute CCl_4_-induced liver injury and EdU incorporation: NGFR (red), PDGFRβ (green), EdU (white), DAPI (blue). Scale bar, 100 μm. Bar plot (right): percentage EdU^+^ PaHSCs versus EdU^+^ CaHSCs (n = 3); error bars SEM. See also Figure S9.

We used the *velocyto* R package (La Manno et al., 2018) to interrogate the HSC injury response by calculating cellular velocity from spliced and unspliced mRNA content. We found that the likelihood of transition between CaHSCs and PaHSCs was negligible, thus inferring absence of pseudotemporal dynamics between the two subpopulations (Figure S9A). Furthermore, fibrogenic genes such as *Col1a1*, *Col3a1*, and *Acta2* display positive residuals (unspliced/spliced mRNA ratio) for CaHSCs but not for PaHSCs, reinforcing their potential for myofibroblast transition (Figure S9B). This demonstrated that HSC differentiation into pathogenic collagen-producing cells occurred in CaHSCs, but not in PaHSCs (Figure 6A). We thus used the *monocle* R package to independently define trajectories for CaHSCs and PaHSCs (Figure 6B). CaHSCs exhibited a branching trajectory (Figure 6B), with one branch transitioning from quiescence into a collagen-producing phenotype with upregulation of pro-fibrogenic genes including *Col1a1*, *Col1a2*, *Col3a1*, and *Acta2*, and the other branch displaying a primarily proliferative response (Figures 6B–6D; Table S3). PaHSCs displayed a proliferative response but did not transition to collagen-producing cells (Figures 6B, 6E, and 6F; Table S3). Immunofluorescence co-staining verified similar levels of proliferation between PaHSCs and CaHSCs following acute CCl_4_-induced liver injury (Figure 6G).

Having identified CaHSCs as the pathogenic collagen-producing cells, we used the *SCENIC* R package (Aibar et al., 2017) to provide mechanistic insight into the transcriptional regulation of HSC activation following both acute and chronic CCl_4_-induced liver injury. *SCENIC* identifies sets of genes that co-express with known transcription factors and are differentially expressed along the CaHSC activation trajectories. We observed 50 such regulons in the acute activation trajectory and 29 in the chronic trajectory of which 18 were shared between both, including *Egr2*, *Sox4*, *Plagl1*, *Rxra*, *Foxf1*, and *Klf7* (Mann and Smart, 2002, Vollmann et al., 2017) (Figures S9C and S9D; Table S3). As both fibroblasts and HSC-derived myofibroblasts are responsible for collagen deposition, we were keen to identify unique regulatory elements for the latter. We identified transcription factors *Sox4* and *Rxra* as specific to HSCs following chronic CCl_4_-induced liver injury (Figure S9E). Again focusing on potential regulatory target genes conserved between the two injury models, *SCENIC* uncovers 6 genes associated with *Rxra* and 5 genes associated with *Sox4* (Figure S9F). Genes associated with the *Sox4* regulon include *Mdk* and *Hmcn1* that have previously been shown to have important roles in fibrosis in other organs (Chowdhury et al., 2014, Misa et al., 2017).

### Targeting of LPAR1 on Collagen-Producing HSCs Inhibits Liver Fibrosis

**Figure 7:**
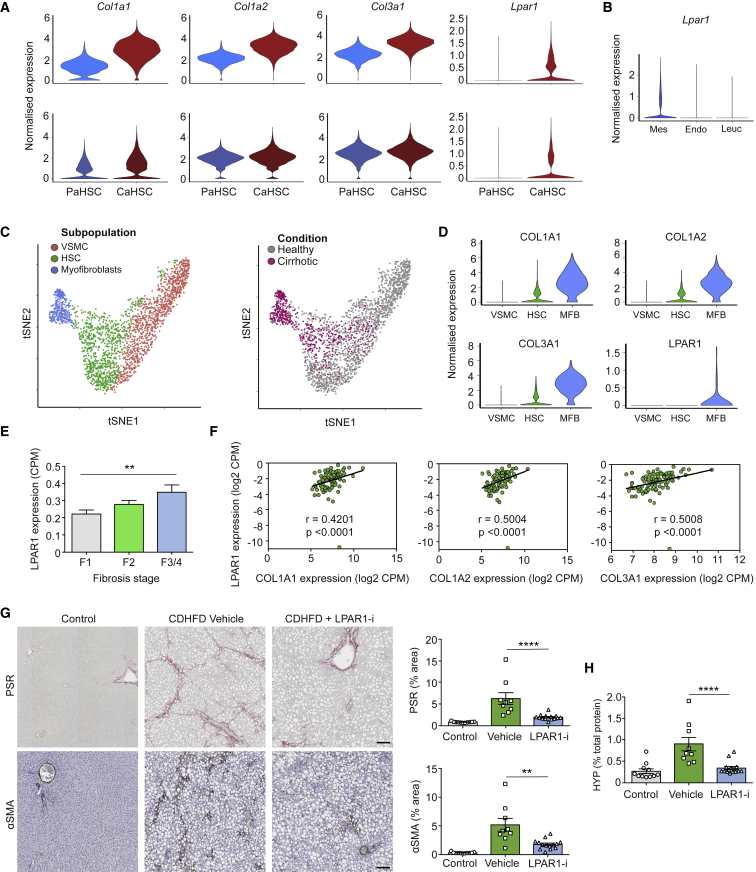
Targeting of LPAR1 on Collagen-Producing HSCs Inhibits Liver Fibrosis (A) Violin plots: expression of fibrillar collagen genes (*Col1a1*, *Col1a2*, and *Col3a1*) and *Lpar1* in PaHSCs versus CaHSCs following acute (72 h post single CCl_4_ injection; top) and chronic (6 weeks CCl_4_; bottom) liver injury. (B) Violin plot: expression of *Lpar1* in hepatic mesenchymal cells (Mes), endothelial cells (Endo), and leucocytes (Leuc) from chronic liver. (C) t-SNE visualization (left): 2,210 mesenchymal cells from healthy and cirrhotic human livers cluster into three subpopulations. t-SNE visualization (right): cells from healthy (gray) and cirrhotic (pink) liver. (D) Violin plots: expression of fibrillar collagen genes (COL1A1, COL1A2, and COL3A1) and LPAR1 across the three human mesenchymal subpopulations. (E) Bar plot: LPAR1 gene expression (RNA-seq) from human FFPE liver samples at different stages of NASH (F1, n = 40; F2, n = 31; F3/4, n = 24); error bars SEM. Kruskal-Wallis test, ^∗∗^p < 0.01. (F) Scatterplot: correlation of LPAR1 expression (y axis) and fibrillar collagen genes (COL1A1, COL1A2, and COL3A1, x axis). r values are Spearman correlation coefficients, p < 0.0001. (G) Representative images of livers from control, CDHFD, and CDHFD + LPAR1 antagonist (LPAR1-i; 30 mg/kg, BID) rodents stained for collagen stain picrosirius red stain (PSR; top) and immunostained for myofibroblast activation marker alpha-smooth-muscle actin (αSMA; bottom). Scale bar, 100 μm. Bar plots (right): percentage area of tissue positive for PSR (top) or αSMA (bottom) (control, n = 11; vehicle, n = 9; LPAR1-i, n = 14); error bars SEM. Mann-Whitney test, ^∗∗^p < 0.01, ^∗∗∗∗^p < 0.0001. (H) Bar plot: hydroxyproline (HYP) levels in liver lysates from control, CDHFD (vehicle), and CDHFD + LPAR1 antagonist (LPAR1-i; 30 mg/kg) rodents (control, n = 11; vehicle, n = 9; LPAR1-i, n = 15); error bars SEM. Mann-Whitney test, ^∗∗∗∗^p < 0.0001. See also Figure S10.

The *Lpar1* gene encodes lysophosphatidic acid receptor 1 (LPAR1), a G protein-coupled receptor that binds the lipid signaling molecule lysophosphatidic acid (LPA). Previous studies have shown LPAR1 expression to be restricted to non-parenchymal cells and elevated in activated HSCs, with minimal expression reported in other hepatic lineages, including hepatocytes (Nakagawa et al., 2016, Simo et al., 2014). Having identified CaHSC as the predominant pathogenic collagen-producing cell during CCl_4_-induced centrilobular murine liver fibrosis, we identified that *Lpar1* was expressed in CaHSC but not PaHSC following acute and chronic CCl_4_-induced liver injury (Figure 7A). *Lpar1* was not expressed in hepatic leucocytes and endothelial cells following chronic CCl_4_-induced liver injury (Figure 7B).

To investigate whether LPAR1 is expressed on pathogenic collagen-producing cells in human liver cirrhosis, we interrogated the hepatic mesenchyme at single-cell resolution in a previously published dataset (Figure 7C) (Ramachandran et al., 2019). Unsupervised clustering identified three mesenchymal subpopulations, including a subpopulation which expands in cirrhosis and is defined by upregulation of fibrillar collagen expression, referred to as myofibroblasts (Figures 7C and 7D). Akin to our observations in murine liver injury, LPAR1 expression was restricted to the collagen-producing subpopulation (Figure 7D).

Previous studies have revealed a role for LPA signaling in HSC activation (Yanase et al., 2000), and LPAR1 antagonism has been shown to reduce fibrosis in a rodent model of hepatocellular carcinoma and mouse model of centrilobular fibrosis (Bollong et al., 2017, Nakagawa et al., 2016). As proof-of-concept of our scRNA-seq approach, we investigated the effects of LPAR1 antagonism on human HSC contractility and activation *in vitro*. LPA, a known ligand of LPAR1, is a major driver of actin polymerization and actomyosin contraction in HSCs (Yanase et al., 2000); LPAR1 antagonism inhibited LPA-induced HSC contractility, pro-fibrogenic connective tissue growth factor (CTGF) expression, F-actin polymerization, and phosphorylation of myosin light chain 2 (Figure S10).

The prevalence of non-alcoholic steatohepatitis (NASH) as a leading cause of chronic liver disease has reached epidemic proportions (Friedman et al., 2018). To assess whether LPAR1 expression increases during the evolution of human NASH-induced liver fibrosis, we performed bulk RNA-seq of human liver samples from a cohort of biopsy-confirmed NASH patients with a range of fibrosis stages (F1–F4). LPAR1 expression increased with fibrosis stage (Figure 7E) and correlated with fibrillar collagen expression (Figure 7F). To further assess whether LPAR1 antagonism inhibits liver fibrosis *in vivo*, we used a 12-week choline-deficient high-fat diet (CDHFD) rodent model of NASH. LPAR1 antagonism markedly reduced liver fibrosis as measured by digital morphometry of picrosirius red and αSMA (Figure 7G) and hydroxyproline assay (Figure 7H).

## Discussion

Despite significant advances in our understanding of the cellular and molecular mechanisms driving liver fibrosis over the past 40 years, there are still no FDA- or EMA-approved antifibrotic treatments currently available. Therefore, there remains a clear imperative to further resolve and understand the complex mechanisms that regulate the fibrotic niche, both in the liver and other organs.

scRNA-seq has facilitated the interrogation of mesenchymal heterogeneity at unprecedented resolution and has greatly advanced our understanding of mesenchymal cell biology and function in disease pathogenesis across various tissues (Croft et al., 2019, Guerrero-Juarez et al., 2019, Peyser et al., 2019, Xie et al., 2018). Investigating specific mesenchymal populations in normal and fibrotic liver has been hampered by the lack of reliable markers required to distinguish these subpopulations (Wells, 2014). In this study, we use a scRNA-seq approach to deconvolve for the first time the entire hepatic mesenchyme in healthy and fibrotic mouse liver, identifying three distinct populations of mesenchymal cells. We make this data freely available to browse at http://livermesenchyme.hendersonlab.mvm.ed.ac.uk, where it should serve as a useful reference resource for future studies of the hepatic mesenchyme.

Multiple mesenchymal cell types have been proposed as the major source of myofibroblasts following liver injury (Iwaisako et al., 2014, Kisseleva et al., 2006, Li et al., 2013, Mederacke et al., 2013), however, recent studies suggest that HSCs are the predominant contributors to the myofibroblast pool irrespective of the cause of liver fibrosis (Iwaisako et al., 2014, Mederacke et al., 2013). Our data confirm HSCs as the dominant mesenchymal contributor to pathogenic collagen production in CCl_4_-induced centrilobular injury.

Zonation across the homeostatic liver lobule has recently been characterized in hepatocytes and endothelial cells using scRNA-seq approaches (Halpern et al., 2017, Halpern et al., 2018), however, zonation of function in the context of a fibrotic injury response has not previously been documented in the liver. HSC zonation has previously been described in porcine liver (Wake and Sato, 1993), and a recent scRNA-seq study in mice concluded that HSCs from healthy liver are a transcriptionally homogeneous population (Krenkel et al., 2019). Furthermore, since the discovery 35 years ago that HSCs are major collagen-producing cells in the liver (Friedman et al., 1985, de Leeuw et al., 1984), their potential to transition to the activated, collagen-secreting myofibroblast phenotype has been thought to be equally distributed across the entire population. In this study, we used scRNA-seq to uncover heterogeneity within the mesenchyme, including zonation of HSCs across the hepatic lobule. We show that HSCs partition into two topographically distinct regions, designated portal vein-associated HSCs (PaHSCs) and central vein-associated HSCs (CaHSCs).

Importantly, we also uncover zonation of function in HSCs, with CaHSCs, but not PaHSCs, responsible for the vast majority of pathogenic fibrillar collagen-production in the CCl_4_ mouse model of centrilobular liver injury. The zonal activation of CaHSCs following induction of CCl_4_ is likely to be secondary to the topographical location of injury, with CCl_4_ causing necrosis of hepatocytes in the centrilobular region (Tanaka and Miyajima, 2016). It is possible that PaHSCs may represent the major collagen-producing HSCs in peri-portal injury models, however, the relative functional roles of PaHSCs and portal fibroblasts in the context of biliary injury requires further investigation (Wells, 2014).

Together, this study provides a high-resolution examination of the hepatic fibrotic niche, via a comprehensive analysis and partitioning of all the hepatic mesenchymal lineages, and investigation of their relative contributions to the fibrogenic process. This scRNA-seq approach has clear implications for the rational development of antifibrotic therapies; facilitating and informing specific targeting of pathogenic scar-forming cells without perturbing homeostatic mesenchymal function, which is of particular importance in patients with chronic liver disease who may already have very limited hepatic functional reserve.

In an era of precision medicine, where molecular profiling underpins the development of highly targeted therapies, we used scRNA-seq to resolve the healthy and fibrotic hepatic mesenchyme in high-definition. Our work illustrates the power of single-cell transcriptomics to identify the key collagen-producing cells driving liver fibrosis with high precision and should also serve as a framework for the high-resolution identification of pathogenic cells and related therapeutic targets in a broad range of fibrotic diseases.

## STAR★Methods

### Key Resources Table

**Table:** 

REAGENT or RESOURCE	SOURCE	IDENTIFIER
**Antibodies**		

Anti-Adamtsl2	Abcam	Abcam Cat# ab97603; RRID:AB_10696110
Anti-Actin, alpha-Smooth Muscle	Biocare	Cat# CM001 [1A1]
Anti-CD31 Platelet Endothelial Cell Adhesion Molecule (PECAM)	BD Biosciences	Cat# 550274; RRID:AB_393571
Anti-CD34	Abcam	Cat# ab81289; RRID:AB_1640331
Anti-Cytokeratin 19	Abcam	Cat# ab220193
Anti-Type I Collagen	SouthernBiotech	Cat# 1310-01; RRID:AB_2753206
Anti- Type III Collagen	SouthernBiotech	Cat# 1330-01; RRID:AB_2794734
Anti-Cyp2e1	Atlas Antibodies	Cat# HPA009128; RRID:AB_1078613
Anti-Calponin 1	Abcam	Cat# ab46794; RRID:AB_2291941
Anti-E-Cadherin	Abcam	Cat# 610181; RRID:AB_397580
Anti-F4/80	Abcam	Cat# ab6640; RRID:AB_1140040
Anti-GFP	Abcam	Cat# ab13970; RRID:AB_300798
Anti-Glutamine Synthetase	Abcam	Cat# ab73593; RRID:AB_2247588
Anti-HNF-4alpha	Santa Cruz Biotechnology	Cat# sc-8987; RRID:AB_2116913
Anti-Lhx2/LH2	Abcam	Cat# ab184337
Anti-MFAP4	Abcam	Cat# ab80319; RRID:AB_1658848
Anti-MYH11	Atlas Antibodies	Cat# HPA015310; RRID:AB_1854261
Anti-p75 NGF Receptor	Abcam	Cat# ab52987; RRID:AB_881682
Anti-Cytokeratin, Wide Spectrum Screening	Agilent	Cat# Z0622; RRID:AB_2650434
Anti-PDGF Receptor beta	Abcam	Cat# ab32570; RRID:AB_777165
Anti-Reelin	R and D Systems	Cat# AF3820; RRID:AB_2253745
Anti-RGS5	Abcam	Cat# ab196799
Anti-Thrombomodulin	Abcam	Cat# ab230010
PE/Cy7 anti-mouse CD45	BioLegend	Cat# 103114; RRID:AB_312979
AF647 anti-mouse CD102	BioLegend	Cat# 105612; RRID:AB_2122182

**Biological Samples**		

Human Hepatic Stellate Cells	Samsara Sciences	Cat #HLSC

**Chemicals, Peptides, and Recombinant Proteins**		

Histodenz	Sigma-Aldrich	Cat#D2158
AM095	DSK Biopharma	N/A, custom order
BMS-986020	MedChem Express	Cat#HY-100619

**Critical Commercial Assays**		

Click-iT Plus EdU Cell Proliferation Kit for Imaging, Alexa Fluor 647 dye	ThermoFisher Scientific	Cat# C10640
RNAscope® Multiplex Fluorescent Reagent Kit v2	Advanced Cell Diagnostics	Cat# 323100
RNAscope® Mm-Adamtsl2-No-XHs	Advanced Cell Diagnostics	Cat# 465521
RNAscope® Mm-Rspo3	Advanced Cell Diagnostics	Cat# 402011
RNAscope® Mm-Col1a1-C2	Advanced Cell Diagnostics	Cat# 31937-C2
Chromium™ Single Cell 3′ Library and Gel Bead Kit	10X Genomics	Cat# PN-120237
Chromium™ Single Cell A Chip Kit	10X Genomics	Cat# PN-120236

**Deposited Data**		

Human mesenchymal cell data	GEO	GSE136103
Mouse mesenchymal cell data	GEO	GSE137720

**Experimental Models: Cell Lines**		

TWNT4	Fuchs et al., 2014	N/A

**Experimental Models: Organisms/Strains**		

*Pdgfrb*-BAC-eGFP	Henderson et al., 2013	N/A

**Software and Algorithms**		

R v3.4.4		http://www.R-project.org
Cell Ranger v2.1.0	10X Genomics	https://www.10xgenomics.com
scater v1.6.3	Bioconductor	McCarthy et al., 2017
scran v1.6.9	Bioconductor	Lun et al., 2016
Seurat v2.3.0	Bioconductor	Satija et al., 2015
SCRAT v1.1.0	http://www.treutleinlab.org	Camp et al., 2017
monocle v2.6.1	Bioconductor	Trapnell et al., 2014
velocyto v0.6.0	Github	La Manno et al., 2018
SCENIC v0.1.7	Bioconductor	Aibar et al., 2017

### Lead Contact and Materials Availability

This study did not generate new unique reagents. Further information and requests for resources and reagents should be directed to and will be fulfilled by the Lead Contact, Neil C. Henderson (Neil.Henderson@ed.ac.uk).

### Experimental Models and Subject Details

#### Mice

*Pdgfrb*-BAC-eGFP reporter mice (on a C57BL/6 background) were obtained from C. Betsholtz. For all experiments, the mice used were 10–16 week old males housed under pathogen–free conditions at the University of Edinburgh. All experiments were performed in accordance with the UK Home Office Regulations.

#### Rats

Wistar-Han rats were obtained from Charles River Laboratories (Kingston). Male 12-week-old rats were used for experiments. All rat experiments were performed in accordance with the Guide for the Care and Use of Laboratory Animals published by the National Research Council (National Academies Press, 2011) and the National Institutes of Health, Office of Laboratory Animal Welfare. Rat studies were run at Covance Laboratories Inc. Greenfield, Indiana, USA.

#### Human tissue

Local approval for procuring human liver tissue for immunofluorescence staining was obtained from the NRS BioResource and Tissue Governance Unit (Study Number SR574), following review at the East of Scotland Research Ethics Service (Reference 15/ES/0094). All subjects provided written informed consent. Healthy background non-lesional liver tissue was obtained intraoperatively from male and female patients undergoing surgical liver resection for solitary colorectal metastasis at the Hepatobiliary and Pancreatic Unit, Department of Clinical Surgery, Royal Infirmary of Edinburgh. Patients with a known history of chronic liver disease, abnormal liver function tests or those who had received systemic chemotherapy within the last four months were excluded from this cohort.

Formalin-fixed, paraffin-embedded (FFPE) liver samples used for tissue RNA-seq were obtained from commercial tissue vendors (Capital Biosciences, Tissue Solutions, and BioIVT); number of samples, n = 95; Gender (Female), n (%), 59 (62%); fibrosis stage, F1,F2,F3/F4 – 40,31,24; NAS score (mean ± SD), 5.2 ± 0.9; age (means ± SD, years), 52.4 ± 11.5).

#### Fibrosis Models

Carbon tetrachloride (CCl_4_) liver injury was induced as described previously (Henderson et al., 2013). For acute CCl_4_-induced liver injury, mice were injected i.p with 1μl/g body weight sterile CCl_4_ in a 1:3 ratio with olive oil (0.25ul/g CCl_4_) after overnight fast (with free access to water); livers were harvested 72 hours post injection. For chronic CCl_4_-induced liver fibrosis mice were injected i.p with 1μl/g body weight CCl_4_ in a 1:3 ratio with olive oil (0.25ul/g CCl_4_) twice weekly for 6 weeks; livers were harvested 48 hours post final injection. To assess proliferation *in vivo*, mice were injected i.p with 5-ethynyl-2′-deoxyuridine (EdU; 50mg/kg; ThermoFisher Scientific, C10640) 3 hours prior to sacrifice.

Nonalcoholic steatohepatisis (NASH) was induced by feeding choline-deficient high-fat diet (CDHFD; Research Diets, Inc., A06071302) or standard chow (LabDiet, 5CR4) to male Wistar-Han rats to induce nonalcoholic steatohepatitis (NASH). Rats were administered LPAR1 antagonist AM095 (Swaney et al., 2011) (30mg/kg) (DSK Biopharma) or vehicle (25% (v/v) PEG 200, 74.625% (v/v) deionized water, 0.375% (w/v) Methyl Cellulose (A4M grade)) twice-daily (BID) via oral gavage while being fed a CDHFD for 12 weeks. Rats were group-housed throughout the experiment and water and feed was provided *ad libitum*.

#### Primary cells and cell lines

Primary human hepatic stellate cells (hHSC) were isolated from viable male livers by density gradient centrifugation (Samsara Sciences; Cat, #HLSC; Donor ID, HL1500002SC). TWNT4 cells were obtained courtesy of Bryan Fuchs, PhD (Fuchs et al., 2014, Nakagawa et al., 2016). TWNT4 cells and hHSC were cultured in DMEM (4.5mg/mL glucose, 110mg/L sodium pyruvate, 4mM L-glutamine) with 10% fetal bovine serum (FBS) supplemented with 100units/mL penicillin and 100mg/mL streptomycin (all from Mediatech, Manassus, VA). Cells were maintained at 37°C in a humidified incubator with 5% CO_2_ in air.

### Method Details

#### Immunofluorescence Staining

Mouse liver was briefly perfused through the inferior vena cava with PBS, then excised. For staining which included intrinsic GFP reporting, tissue was fixed/frozen. For all other staining, tissue was formalin-fixed paraffin-embedded.

##### Fixed/frozen sections

Liver was fixed in 4% paraformaldehyde for 2 hours at 4°C then immersed in graded sucrose solutions, embedded in OCT and stored at –80°C. 7μm frozen sections were cut and left to air dry for 30 minutes, washed in PBS, then blocked using protein block (GeneTex, GTX30963) for 30 minutes. Sections were then incubated with antibodies listed in Table S4 overnight at 4°C. Following a further PBS wash sections were incubated with fluorescently conjugated secondary antibodies depending on host species (Alexa Fluor 555 goat anti-rat; Alexa Fluor 555 goat anti-rabbit; Alexa Fluor 555 donkey anti-mouse; Alexa Fluor 488 goat anti-chicken (Life Technologies, A21434, Lot. 1722994; A21429, Lot.1937155; A31570, Lot.1850121; A11039; Lot.1869581, respectively). Co-stains were completed sequentially. Slides were washed further in PBS before DAPI-containing mountant was applied (ThermoFisher Scientific, P36931). For PDGFRβ staining, before blocking, heat-mediated antigen retrieval in pH9 Tris-EDTA (microwave; 2 minutes) was performed. Slides were washed in PBS, incubated in 3% hydrogen peroxide for 10 minutes and washed again in PBS before proceeding with the above protocol. Instead of incubating with a fluorescently conjugated secondary antibody sections were washed with PBS and then incubated with ImmPress HRP Polymer Detection Reagents (rabbit, MP-7401 Vector Laboratories) for 30 minutes and washed in PBS again. Staining was detected using Cy3 tyramide (Perkin-Elmer, NEL744B001KT) at 1:1000 dilution. Sections were imaged using a slide scanner (AxioScan.Z1, Zeiss) at 20X magnification. Images were processed using Zen Blue (Zeiss) and Fiji image software.

##### Formalin-fixed paraffin-embedded (FFPE) sections:

Liver was fixed in 4% neutral-buffered formalin for 24 hours followed by paraffin-embedding. 5μm sections were cut, dewaxed, rehydrated, then incubated in 4% neutral-buffered formalin for 20 minutes. Following heat-mediated antigen retrieval in pH6 sodium citrate or pH9 Tris-EDTA (microwave; 15minutes), slides were washed in PBS and incubated in 3% hydrogen peroxide for 10 minutes. Slides were then washed in PBS, blocked using protein block (GeneTex, GTX30963) for 1 hour at room temperature, and incubated with primary antibodies. A full list of primary antibodies and conditions are shown in Table S4. Slides were then washed in PBS/T (PBS plus 0.1% Tween 20; Sigma-Aldrich, P1379), incubated with ImmPress HRP Polymer Detection Reagents (depending on species of primary; rabbit, MP-7401; mouse, MP-6402-15; goat, MP-7405; all Vector Laboratories) for 30 minutes at room temperature, and washed again with PBS/T. Staining was detected using either Cy3, Cy5, or Fluorescein tyramide (Perkin-Elmer, NEL741B001KT) at 1:1000 dilution. For multiplex stains slides were then washed in PBS/T followed by a further heat treatment with pH6 sodium citrate or pH9 Tris-EDTA (15 minutes), washed in PBS, incubated in 3% hydrogen peroxide for 10 minutes, washed in PBS, protein blocked, and finally incubated with the second primary antibody followed by the ImmPress Polymer and tyramide as before. When required this sequence was repeated for the third primary antibody. A DAPI-containing mountant was then applied (ThermoFisher Scientific, P36931). Sections were imaged using a slide scanner (AxioScan.Z1, Zeiss) at 20X magnification. Images were processed using Zen Blue (Zeiss) and Fiji image software.

αSMA (Biocare CM001 [1A1], Ms mAb, 0.12ug/mL) IHC staining of rat liver was performed using the Biocare Intellipath autostainer utilizing Biocare Medical reagents. Following deparaffinization, slides were sequentially treated with hydrogen peroxidase for 5 minutes, citrate-based heat induced (95°C) antigen retrieval for 40 minutes, protein block for 10 minutes, primary antibody for 30 minutes, one-step polymer-HRP conjugated (mouse on rat HRP) secondary antibody for 30 minutes, DAB chromogen for 5 minutes, CAT Hematoxylin for 5 seconds, and bluing solution for 10 seconds.

#### RNAscope

Detection of *Adamtsl2*, *Rspo3*, and *Col1a1* was performed using the RNAscope® Multiplex Fluorescent Reagent Kit v2 (Advanced Cell Diagnostics (ACD), Cat, 323100) in accordance with the manufacturer’s instructions. Briefly, 5μm liver sections were dewaxed, incubated with endogenous enzyme block, boiled in pretreatment buffer and treated with protease, followed by target probe hybridization using the RNAscope® Mm-Adamtsl2-No-XHs (Cat, 465521; Lot, 19086B; ACD), Mm-Rspo3 (Cat, 402011; Lot, 18338A; ACD) or Mm-Col1a1-C2 (Cat, 31937-C2; Lot, 19086C; ACD) probes. Target RNA was detected with Cy3 (*Adamtsl2* or *Rspo3*) or Fluorescein (*Col1a1*) tyramide (Perkin-Elmer) at 1:750 dilution. For combined RNAscope and immunofluorescence staining sections were processed as for multiplex immunofluorescence staining (as above) after the RNAscope protocol. Slides were imaged using a slide scanner (AxioScan.Z1, Zeiss) at 40X (for RNAscope) magnification or a Zeiss LSM780 inverted confocal microscope. Images were processed using Zen Blue (Zeiss) and Fiji image software.

#### EdU Click-iT Immunofluorescence staining

EdU incorporation into DNA was detected using the Click-iT EdU Alexa Fluor Imaging kit (Invitrogen/Molecular Probes, C10640). Formalin-fixed paraffin-embedded 5μm sections were dewaxed, rehydrated, then incubated in 4% neutral-buffered formalin for 20 minutes. Sections were washed in 0.1% Triton X-100 in PBS (PBSTX) for 10 minutes followed by heat-mediated antigen retrieval in pH6 sodium citrate (microwave; 15 minutes), washed for 10 minutes in PBSTX and blocked for 1 hour using protein block (GeneTex, GTX30963). The Click-iT solution was then made according to manufacturer’s instructions. Slides were incubated in the EdU cocktail for 30 minutes and rinsed three times in PBS. The azide used was coupled to an Alexa Fluor 647 fluorophore. Upon completion of the EdU Click-iT reaction, slides were processed as above for multiplex staining. Sections were imaged using a slide scanner (AxioScan.Z1, Zeiss) at 20X magnification. Images were processed using Zen Blue (Zeiss) and Fiji image software.

#### Picrosirius Red Staining

Picrosirius red (PSR) staining was performed using 0.1% Direct Red 80 (Sigma) in 1.3% picric acid solution (Sigma, 239801). Formalin-fixed paraffin-embedded 5μm sections were dewaxed, rehydrated, incubated in 0.4% phosphomolybdic acid for 5 minutes, and washed with PBS. Sections were then stained with picosirius red for two hours, before washing twice with agitation for 30 s in acidified water. Slides were placed in 0.1% Fast Green (ThermoFisher Scientific, F/P025/46) for 30 seconds followed by two 30 seconds washes with agitation in acidified water. Following dehydration (100% ethanol), slides were cleared in xylene and mounted using DPX. Sections were imaged using a slide scanner (AxioScan.Z1, Zeiss) at 20X magnification.

#### Image Quantification

Cell counts for zonation of HSC in uninjured liver, Lhx2^+^ cell expansion, zonation of HSC following acute liver injury, *Pdgfrb*-BAC-eGFP reporting efficiency and specificity, and NGFR and *Adamtsl2* marker specificity were counted manually from multiple high-powered images per sample. For zonation in uninjured liver the peri-central and peri-portal regions were defined as areas of positive Cyp2e1 or E-cadherin staining, respectively. All areas were processed using Zen Blue software to calculate cell count/mm^2^. In uninjured liver and following acute liver injury (72 hours post single CCl_4_) NGFR was used as a marker for PaHSC and *Adamtsl2* as a marker for CaHSC. For quantification of proliferation following acute injury PaHSC were identified as NGFR^+^/PDGFRβ^+^ cells and CaHSC as NGFR^-^/PDGFRβ^+^ cells.

To quantify PSR staining digital morphometric pixel analysis was performed using the Trainable Weka Segmentation (TWS) plugin (Arganda-Carreras et al., 2017) in Fiji software (Schindelin et al., 2012). Briefly, the TWS plugin was trained to produce a classifier segmenting images into areas of positive staining, tissue background and white space. The same trained classifier was applied to all images to produce a percentage area of positive staining for each tissue section.

Quantitative image analysis of PSR staining in rat tissue was performed using Visiopharm v2017.2. The Tissue Find APP was used to find the tissue regions in the images, before positive expression was categorized as low, medium, or high based on the level of staining intensity. Percentage area was calculated by summing the values for low, medium, and high expression, dividing by the value for staining on the entire tissue area, and multiplying by 100.

#### Hydroxyproline Assay

Liver samples were dehydrated overnight at 62°C, followed by homogenization in water (50μL water/1mg dry tissue weight) using a bead-based TissueLyser. Total protein was measured using a BCA protein assay (Pierce (Thermo Fisher) BCA Protein Assay Kit; 23227). Homogenates were hydrolyzed overnight in 6 N HCl at 110°C. Samples and orthohydroxyproline standards were added in duplicate to microplate wells and dried. Chloramine T (Sigma; 857319) was added to all wells and the plate was incubated at room temperature for 30 minutes with shaking. Ehrlich’s Reagent (Fisher; D71-25) was added to all wells and the plate sealed and incubated at 60°C for 40 minutes. Optical density was measured at 560nm on Molecular Devices’ SpectraMAX PLUS Microplate Reader. OH-P content was calculated for all samples and normalized to total protein and compared to an 8-point standard curve.

#### Immunocytochemistry

TWNT4 cells (Nakagawa et al., 2016) (courtesy of Bryan Fuchs, PhD) were plated on 96-well optical plates (Greiner Bio-One; 655946) in complete media (DMEM, (GIBCO; 15-018-CM), 10% FBS, (Hyclone; SH30088.03), Penicillin-streptomycin-glutamate (GIBCO; 10378)), serum-starved overnight, was pretreated with DMSO or 1μM LPAR1 antagonist (BMS-986020) (Palmer et al., 2018) (Medchem Express; HY-100619) for 30 minutes, then treated with 0.1% BSA (control) or 10μM 18:2 LPA (Avanti Polar Lipids; 857138) for 20 minutes. Cells were then fixed in 4% paraformaldehyde for 20 minutes at room temperature, washed with PBS, permeabilized with 0.3% TritionX-100/PBS, blocked with 2% BSA/PBS, and stained with Alexa647-phalloidin, mouse anti-pMLC2 (mAb3675, Cell Signaling Technology), and Hoechst 33342 (ThermoFisher; H3570). Images were acquired with ImageXpress Pico automated imaging system (Molecular Devices).

#### Contraction Assay

Collagen gel contraction assay was performed using the CytoSelect 48-Well Cell Contraction Assay Kit (CBA-5021). In brief, TWNT4 cells (200K cells/well) were mixed with collagen solution and allowed to polymerize at 37°C in a CO_2_ incubator for 1 hour. After collagen gel polymerization, 0.5ml media (Serum free- DMEM, (GIBCO; 15-018-CM)) containing either 3 μM LPAR1 antagonist (BMS-986020) or DMSO were added atop the collagen lattice for 30 minutes, followed by addition of 50 μM LPA (Avanti Polar Lipids) and incubation at 37°C and 5% CO_2._ Media was changed daily by carefully removing 250μl of media and replacing with 250μl (with /without contraction mediators)_._ Collagen gel contraction was measured after 96 hours using light inverted microscopy or on a Celigo imaging cytometer platform (Nexcelom Biosciences) using the bright-field channel. Contracted gel area was quantified using ImageJ analysis.

#### qPCR

Primary HSC or TWNT4 cells were pre-treated for 30 minutes with 3μM LPAR1 antagonist (BMS-986020) or DMSO, followed by addition of 10μM 18:2 LPA (Avanti Polar Lipids) for 2 hours. RNA was isolated using the RNeasy Plus Mini Kit (QIAGEN) and cDNA was made using the High Capacity cDNA Reverse Transcription Kit (ThermoFisher Scientific; 4368814). RT-qPCR for CTGF and HPRT1 was performed using TaqMan Gene Expression Assays (ThermoFisher Scientific, Hs00170014_m1 and Hs02800695_m1).

#### Primary cell isolation

##### Digestion protocol 1

Mouse liver was perfused through the inferior vena cava with phosphate buffered saline (PBS). The liver was excised, minced with a scalpel, digested in 5mg/ml pronase (Sigma, P5147), 2.84mg/ml collagenase B (Roche, 11088815001; 0.188U/mg) and 0.019mg/ml DNase 1 (Roche, 10104159001) at 37°C for 20 minutes with agitation (200–250 rpm), and then strained through a 120μm nybolt mesh. The cell suspension was centrifuged at 400*g* for 7 minutes, supernatant removed, cell pellet resuspended in PEB buffer (PBS, 2% FBS, and 2mM EDTA), and DNase I added (0.02mg/ml). Following red blood cell lysis with RBC lysis buffer (BioLegend; Cat:420301), the cell suspension was again centrifuged at 400*g* for 7 minutes, supernatant removed, cell pellet resuspended in PEB buffer and DNase 1 added (0.02mg/ml).

##### Digestion protocol 2

Hepatic stellate cells (HSC) were isolated from mice as described previously (Mederacke et al., 2015). Mice were anaesthetized via inhalation of isoflurane (1%–3%). Following cannulation of the inferior vena cava, the portal vein was cut to allow retrograde stepwise perfusion of EGTA (0.19mg/ml; 2 minutes), pronase (0.4mg/ml; 5 minutes; Sigma, P5147) and collagenase D (0.185U/ml; 7 minutes; Roche, 11088882001) containing GBSS/B solutions (Sigma, G9779). Liver was then excised and minced before *ex vivo* digestion in GBSS/B (Sigma, G9779) containing 0.5mg/ml pronase, 0.088U/ml collagenase D and 1% DNase 1 (Roche, 10104159001). The resulting cell suspension was then strained through a 70μm cell strainer and centrifuged at 580g for 10 minutes, before supernatant was removed and the cells resuspended in GBSS/B containing DNase I. Following a further centrifugation (580g for 10 minutes), HSC were isolated from the digest solution by Histodenz (Sigma, D2158-100G) gradient centrifugation (1380g for 17 minutes).

#### Cell sorting

Cells were blocked in 1% purified anti-mouse CD16/32 (BioLegend; Clone. 93 Cat. 101324; Lot. B254979) and 10% normal mouse serum (Sigma, M5905) for 10 minutes at 4°C before incubation with antibodies CD45-PE/Cy7 (1:100; BioLegend; Clone: 30-F11; Cat. 103114; Lot. B243728) and CD102-AF647 (1:100; BioLegend; Clone: 3C4 (MIC2/4); Cat. 105612; Lot. B227625) for 20 minutes at 4°C. For cells isolated from digestion protocol 1 live/dead (DAPI 1:1000) staining was performed immediately prior to running the samples. For cells isolated from digestion protocol 2 DAPI was replaced with 7-AAD viability stain (BioLegend; Cat. 420404; Lot. B251165). Cell sorting was performed on a FACS Aria II (Becton Dickinson, Basel, Switzerland).

#### Human liver tissue RNA-seq

RNA sequencing was performed by Q2 Solutions (Morrisville, North Carolina). For human liver samples, total RNA was isolated from three 11μm FFPE curls per sample in one tube (33μm total). All samples had > 100ng of input RNA. Sequencing libraries were created using the TruSeq RNA Access target enrichment and library preparation methodology which provides high data quality data even from degraded or FFPE-derived RNA samples. Libraries were sequenced on a Illumina HiSeq2500 with 50bp paired-end sequencing and a total read depth of 40M reads per sample. R packages *edgeR* and *limma* were used to normalize sequence count data and conduct differential gene-expression analysis. False discovery rate (FDR) was calculated using the Benjamini-Hochberg method (Benjamini and Hochberg, 1995).

#### Single-cell workflows

##### 10X Chromium

Single cells were processed through the Chromium™ Single Cell Platform using the Chromium™ Single Cell 3′ Library and Gel Bead Kit v2 (10X Genomics, PN-120237) and the Chromium™ Single Cell A Chip Kit (10X Genomics, PN-120236) as per the manufacturer’s protocol. In brief, single cells were sorted into PBS + 2% FBS, washed twice and counted using a Bio-Rad TC20. Approxiamtely 10,769 cells were added to each lane of a 10X chip and partitioned into Gel Beads in Emulsion in the Chromium™ instrument, where cell lysis and barcoded reverse transcription of RNA occurred, followed by amplification, fragmentation and 5′ adaptor and sample index attachment. Libraries were sequenced on an Illumina HiSeq 4000.

##### Smart-seq2

Single cells were processed by SciLifeLab – Eukaryotic Single cell Genomic Facility (Karolinska Institute). Before shipping single cells were sorted into wells of a 384-well plate containing pre-prepared lysis buffer. Libraries were sequenced on an Illumina HiSeq 4000.

#### Pre-processing scRNA-seq data

##### Mouse 10X Chromium:

We aligned to the mm10 reference genome (Ensembl 84) and estimated cell-containing partitions and associated UMIs using the Cell Ranger v2.1.0 Single-Cell Software Suite from 10X Genomics. Genes expressed in fewer than three cells in a sample were excluded, as were cells that expressed fewer than 300 genes or mitochondrial gene content > 30% of the total UMI count. We normalized by dividing the UMI count per gene by the total UMI count in the corresponding cell and log-transforming. Variation in UMI counts between cells was regressed according to a negative binomial model, prior to scaling and centering the resulting value by subtracting the mean expression of each gene and dividing by its standard deviation (E_n_), then calculating ln(10^4^^∗^E_n_+1). Highly variable genes were identified using Seurat’s *FindVariableGenes* function with default parameters. Non-mesenchymal mouse scRNA-seq data following chronic CCl_4_-induced liver fibrosis were analyzed from our previously-obtained datasets (Ramachandran et al., 2019).

##### Human 10X Chromium:

We aligned to the GRCh38 reference genome (Ensembl 84) and processed our single-cell transcriptomic data as above. Mesenchymal cells were isolated based on PDGFRB expression.

##### Mouse Smart-seq2

The single-cell transcriptomic data was initially processed at the Eukaryotic Single-Cell Genomics Facility at the Science for Life Laboratory in Stockholm, Sweden: obtained reads were mapped in STAR to the mm10 build of the mouse genome (concatenated with transcripts for eGFP and the ERCC spike-in set), and then processed via rpkmforgenes, MULTo, and RefSeq to yield a count for each endogenous gene, spike-in, and eGFP transcript per cell. We performed quality control in R packages *scater* v1.6.3 (McCarthy et al., 2017) and *scran* v1.6.9 (Lun et al., 2016), removing cells with library size or features less than, or with ERCC percentage greater than, 3 median absolute deviations from the dataset median. We then computed normalized expression using sum factors (with separate calculation of spike-in factors), before transferring these values to Seurat to identify highly variable genes as above.

#### Dimensionality reduction, clustering, and DE analysis

We performed unsupervised clustering and differential gene expression analyses in the *Seurat* R package v2.3.0 (Satija et al., 2015). In particular we used SNN graph-based clustering, where the SNN graph was constructed using from 2 to 10 principal components as determined by dataset variability shown in principal components analysis (PCA); the resolution parameter to determine the resulting number of clusters was also tuned accordingly. In total, we present scRNA-seq data from nine mouse liver samples in 10X (three uninjured, three acute CCl_4_, three chronic CCl_4_) and three mouse liver samples in SmartSeq2 (all uninjured).

All heatmaps, t-SNE visualizations, and violin plots were produced using *Seurat* functions in conjunction with the *ggplot2*, *pheatmap*, and *grid* R packages. t-SNE visualizations were constructed using the same number of principal components as the associated clustering, with perplexity ranging from 100 to 300 according to the number of cells in the dataset. We conducted differential gene expression analysis in *Seurat* using an AUC classifier to assess significance, retaining only those genes with a log-fold change of at least 0.25 and expression in at least 25% of cells in the cluster under comparison.

#### Defining cell expression signatures

Signature scores were defined per cell as the geometric mean of the expression of the associated signature genes, scaled to a range of 0 to 1 across the dataset. For a signature of fibrillar collagen production we aggregated expression of the following genes: *Col1a1*, *Col1a2*, *Col3a1*. For a signature of proliferation we aggregated expression of the following genes: *Mki67*, *Cdca8*, *Cdc20*, *Ccna2*, *Ccnb1*. To perform unbiased thresholding on these scores we used k-means clustering (using the *threshold* function from the *mmand* R package) and binarised the results.

#### Identifying and applying an HSC zonation signature

We used unsupervised Independent Component Analysis (ICA) in *Seurat* to generate components of variability in uninjured HSCs. Analyzing each component in turn, we identified and isolated the one with highest correlation to observed *Ngfr* heterogeneity. We then thresholded on the gene weight loadings along this component of interest to extract an 81-gene signature, including 52 genes associated with *Ngfr* and 29 genes associated with *Spon2*.

Using this 81-gene signature as the input to supervised Seurat clustering, we clustered the homeostatic HSCs into two subpopulations: *Ngfr*^hi^ and *Ngfr*^lo/neg^. We classified acute and chronic CCl_4_ HSCs into the same two subpopulations in the same manner. To assess whether the zonation profiles of these signature genes remained consistent across acute and chronic injury, we ordered the cells at each time point by the strength at which they expressed the 52-gene *Ngfr*-associated signature and manually inspected the profile of each gene across this ordering.

#### Analyzing functional phenotypes of mesenchymal cells

For further analysis of the function related gene expression profile we adopted the self-organizing maps (SOM) approach as implemented in the *SCRAT* R package v1.0.0^27^. For each lineage of interest we constructed a SOM in *SCRAT* using default input parameters and according to its clusters. We defined the signatures expressed in a cell by applying a threshold criterion (e_thresh_ = 0.95 × e_max_) selecting the highest-expressed metagenes in each cell, and identified for further analysis those metagene signatures defining at least 30% of cells in at least one cluster within the lineage. We smoothed these SOMs using the *disaggregate* function from the *raster* R package for visualization purposes, and scaled radar plots to maximum proportional expression of the signature. Gene ontology enrichment analysis on the genes in these spots was performed using PANTHER 13.1 (http://pantherdb.org).

#### Inferring injury dynamics and transcriptional regulation

To generate cellular trajectories (pseudotemporal dynamics) we used the *monocle* R package v2.6.1 (Trapnell et al., 2014). We ordered cells (*Ngfr*^hi^ versus *Ngfr*^lo/neg^) in an unsupervised manner, scaled the resulting pseudotime values from 0 to 1, and mapped these onto the t-SNE visualizations generated by *Seurat*. We removed mitochondrial and ribosomal genes from the geneset for the purposes of trajectory analysis. Differentially-expressed genes along this trajectory were identified using generalized linear models via the *differentialGeneTest* function in *monocle*.

When determining significance for differential gene expression along the trajectory, we set a q-value threshold of 1e^-20^. We clustered these genes using hierarchical clustering in *pheatmap*, cutting the tree at k = 3 to obtain gene modules with correlated gene expression across pseudotime. Cubic smoothing spline curves were fitted to scaled gene expression along this trajectory using the *smooth.spline* command from the *stats* R package, and gene ontology enrichment analysis again performed using PANTHER 13.1.

We verified the trajectory and its directionality using the *velocyto* R package v0.6.0^35^, estimating cell velocities from their spliced and unspliced mRNA content. We generated annotated spliced and unspliced reads from the 10X BAM files via the *dropEst* pipeline, before calculating gene-relative velocity using kNN pooling with k = 25, determining slope gamma with the entire range of cellular expression, and fitting gene offsets using spanning reads. Aggregate velocity fields (using Gaussian smoothing on a regular grid) and transition probabilities per lineage subpopulations were visualized on t-SNE visualizations as generated previously. Gene-specific phase portraits were plotted by calculating spliced and unspliced mRNA levels against steady-state inferred by a linear model; levels of unspliced mRNA above and below this steady-state indicate increasing and decreasing expression of said gene, respectively. Similarly we plotted unspliced count signal residual per gene, based on the estimated gamma fit, with positive and negative residuals indicating expected upregulation and downregulation respectively.

For transcription factor analysis, we obtained a list of all genes identified as acting as transcription factors in humans from AnimalTFDB (Zhang et al., 2015). To further analyze transcription factor regulons, we adopted the *SCENIC* v0.1.7 workflow in R (Aibar et al., 2017), using default parameters and the normalized data matrices from *Seurat* as input. For visualization, we mapped the regulon activity (AUC) scores thus generated to the pseudotemporal trajectories from monocle and the clustering subpopulations from *Seurat*.

### Quantification and Statistical Analysis

Statistical analyses were performed using GraphPad Prism (GraphPad Software, USA). Comparison of changes in histological cell counts, topographical localization of counted cells, morphometric pixel analysis, and gene expression were performed using a Mann-Whitney test (unpaired; two-tailed). Comparison of RNA-seq data from human NASH patients was performed using a Kruskal-Wallis test with Dunn’s multiple comparisons test. Analysis of correlation was performed using a Spearman correlation coefficient. All statistical tests used, exact value of n, and P values obtained are displayed in the figure legends. P values < 0.05 were considered statistically significant.

### Data and Code Availability

All mouse mesenchymal data is deposited in the Gene Expression Omnibus. The accession number for the data is GEO: GSE137720. All human mesenchymal data, as well as mouse leucocyte data, is available from the Gene Expression Omnibus (GEO: GSE136103).

R markdown scripts enabling the main steps of the analysis are available from the Lead Contact upon reasonable request.

#### Additional Resources

Our uninjured and 6 week CCl_4_ expression data is freely available for user-friendly interactive browsing online: http://livermesenchyme.hendersonlab.mvm.ed.ac.uk

## Supplemental Information

Document S1. Figures S1–S10

Table S1. Marker and Zonation Genes, Related to Figures 1, 2, 3, 4, and 5 This table provides the list of marker genes specific to each of the identified clusters in the homeostatic murine hepatic mesenchyme dataset (Figure 1B), the homeostatic murine hepatic mesenchyme SmartSeq2 dataset (Figure S3A), and the homeostatic and fibrotic murine hepatic mesenchyme dataset (Figure 3A). It also provides the list of genes used as zonation signatures for analysis (Figures 2A, 4A, and S8F).

Table S2. Metagene Signatures and Associated Ontology Terms, Related to Figures 1 and 3 This table provides the list of genes associated with each SOM metagene signature identified as distinguishing subpopulations within the homeostatic murine hepatic mesenchyme dataset (Figures 1F and S2F), the homeostatic murine hepatic mesenchyme SmartSeq2 dataset (Figure S3E), and the homeostatic and fibrotic murine hepatic mesenchyme dataset (Figure S7C), as well as their associated gene ontology terms.

Table S3. Gene Modules and Associated Ontology Terms and Transcription Factor Regulons across Pseudotemporal Trajectories, Related to Figures 4 and 6 This table provides the list of genes associated with each module of differentially expressed genes over the central-associated HSC pseudotemporal trajectory in homeostatic and fibrotic murine hepatic mesenchyme (Figures 4E and 4F), the central-associated HSC pseudotemporal trajectory in acute-injury murine hepatic mesenchyme (Figures 6C and 6D), and the portal-associated HSC pseudotemporal trajectory in acute-injury murine hepatic mesenchyme (Figures 6E and 6F), as well as their associated GO terms. It also provides the list of transcription factor regulons differentially expressed over the central-associated HSC pseudotemporal trajectory in homeostatic and fibrotic murine hepatic mesenchyme and the central-associated quiescent to activated HSC pseudotemporal trajectory in acute-injury murine hepatic mesenchyme (Figures S9C and S9D).

Table S4. Antibodies Used for Immunofluorescence, Related to STAR Methods This table provides a list of commercial antibodies and conditions used in this study (STAR Methods).

Document S2. Article plus Supplemental Information
