# Supplementary material for: Single-Cell Transcriptomics Uncovers Zonation of Function in the Mesenchyme during Liver Fibrosis
Source: Cell Rep. 2019 Nov 12;29(7):1832–1847.e8. doi: 10.1016/j.celrep.2019.10.024 (PMC6856722; doi:10.1016/j.celrep.2019.10.024)
Supplement: Document S1. Figures S1–S10 [file mmc1.pdf]

Cell Reports, Volume *29*

Supplemental Information

Single-Cell Transcriptomics Uncovers Zonation of Function in the Mesenchyme during Liver Fibrosis

Ross Dobie, John R. Wilson-Kanamori, Beth E.P. Henderson, James R. Smith, Kylie P. Matchett, Jordan R. Portman, Karolina Wallenborg, Simone Picelli, Anna Zagorska, Swetha V. Pendem, Thomas E. Hudson, Minnie M. Wu, Grant R. Budas, David G. Breckenridge, Ewen M. Harrison, Damian J. Mole, Stephen J. Wigmore, Prakash Ramachandran, Chris P. Ponting, Sarah A. Teichmann, John C. Marioni, and Neil C. Henderson

Supplemental Figure S1

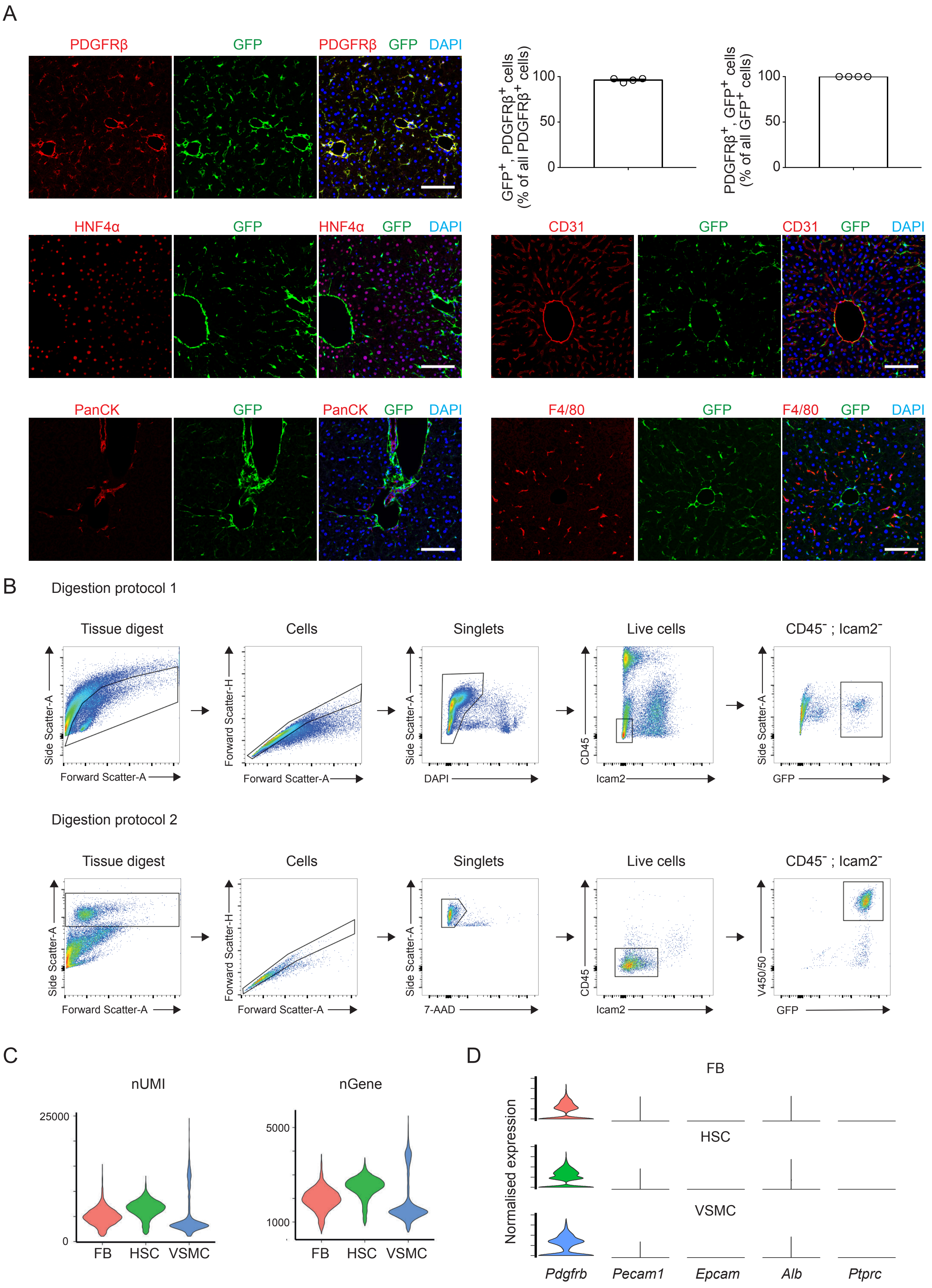

Supplemental Figure S1: Isolation of liver mesenchymal cells from *Pdgfrb*-BAC-eGFP reporter mice, related to Figure 1

(A) Representative immunofluorescence images of healthy murine liver: PDGFRβ / HNF4α / PanCK / CD31 / F4/80 (red), GFP (green), DAPI (blue). Scale bar, 100µm. Bar plots (top right): specificity and efficiency of *Pdgfrb*-BAC-eGFP reporting (n=4); error bars SEM. (B) Representative flow cytometry plots: gating strategies following isolation of mesenchymal cells from healthy murine liver digested using digestion protocol 1 or 2 (Methods). (C) Violin plots: number of total Unique Molecular Identifiers (nUMI) and number of unique genes (nGene) expressed in mesenchymal cells from healthy murine liver. (D) Violin plots: expression of mesenchymal and non-mesenchymal cell lineage markers in the healthy liver mesenchyme dataset (*Pecam1*: CD31; *Ptprc*: CD45).

Supplemental Figure S2

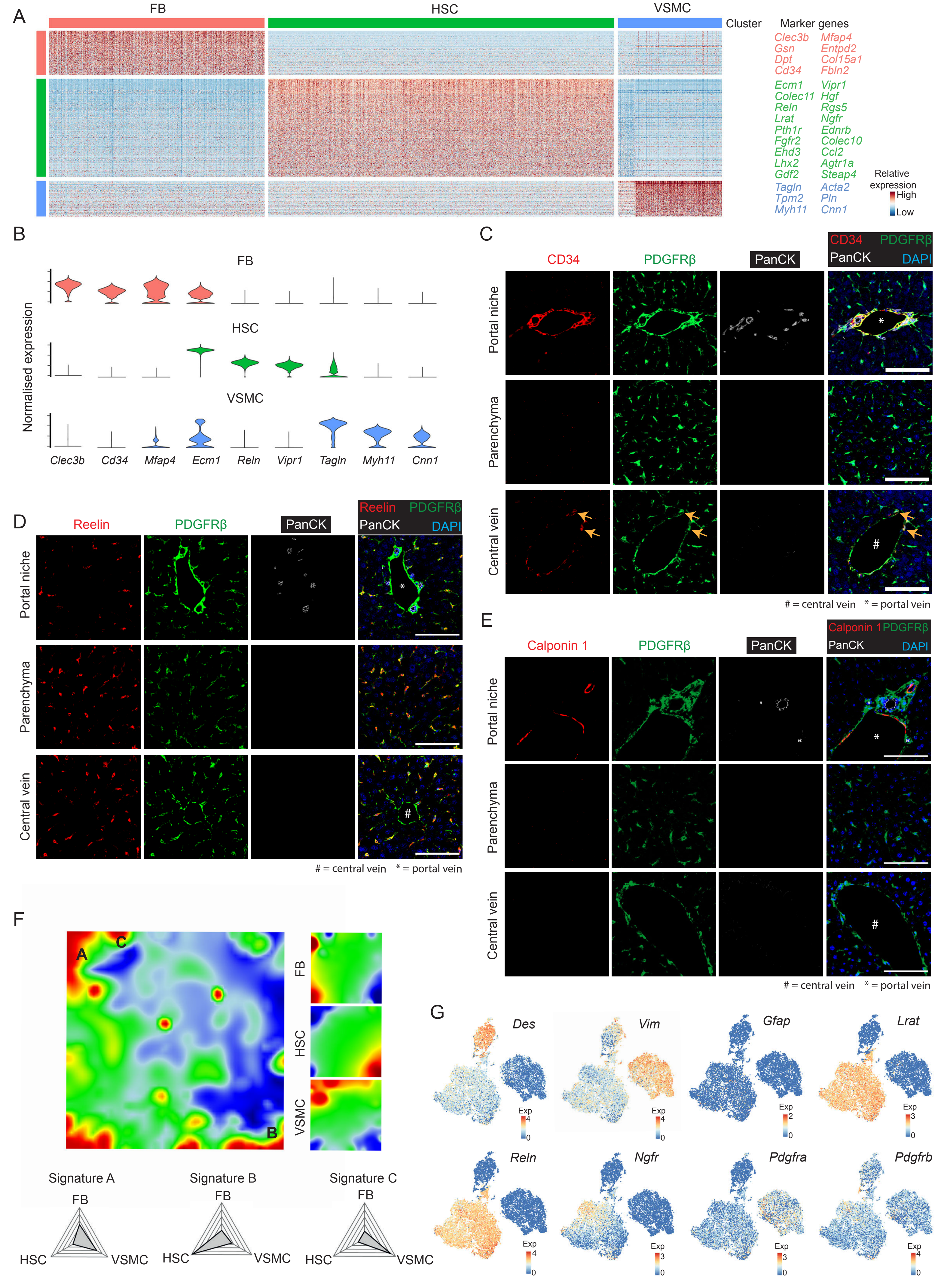

Supplemental Figure S2: Deconvolution of the mouse hepatic mesenchyme identifies three distinct subpopulations in liver homeostasis, related to Figure 1

(A) Heatmap of relative expression: cluster marker genes (coloured by subpopulation) with exemplar genes labelled (right). Cells columns, genes rows. (B) Violin plots: exemplar markers for the three subpopulations. (C) Representative immunofluorescence images of healthy murine livers focussing on portal niche, parenchyma and central vein: CD34 (red), PDGFRβ (green), PanCK (white), DAPI (blue). Scale bar, 100μm; portal vein (*) and central vein (#) as indicated. Yellow arrows indicate $CD34^{+}$ $PDGFR\beta^{+}$ cells around the central vein. (D) Representative immunofluorescence images of healthy murine livers focussing on portal niche, parenchyma and central vein: Reelin (red), PDGFRβ (green), PanCK (white), DAPI (blue). Scale bar, 100μm; portal vein (*) and central vein (#) as indicated. (E) Representative immunofluorescence images of healthy murine livers focussing on portal niche, parenchyma and central vein: Calponin 1 (red), PDGFRβ (green), PanCK (white), DAPI (blue). Scale bar, 100μm; portal vein (*) and central vein (#) as indicated. (F) Self-Organising Map (SOM; 60x60 grid): smoothed scaled metagene expression in healthy murine mesenchyme. 16,421 genes, 3,600 metagenes, 14 signatures. A-C label metagene signatures overexpressed in one or more of the subpopulations (right). Radar plots (below): distribution of metagene signature A-C expression across the three subpopulations. (G) t-SNE visualisations: traditional mesenchymal cell and HSC marker gene expression in the healthy dataset.

Supplemental Figure S3

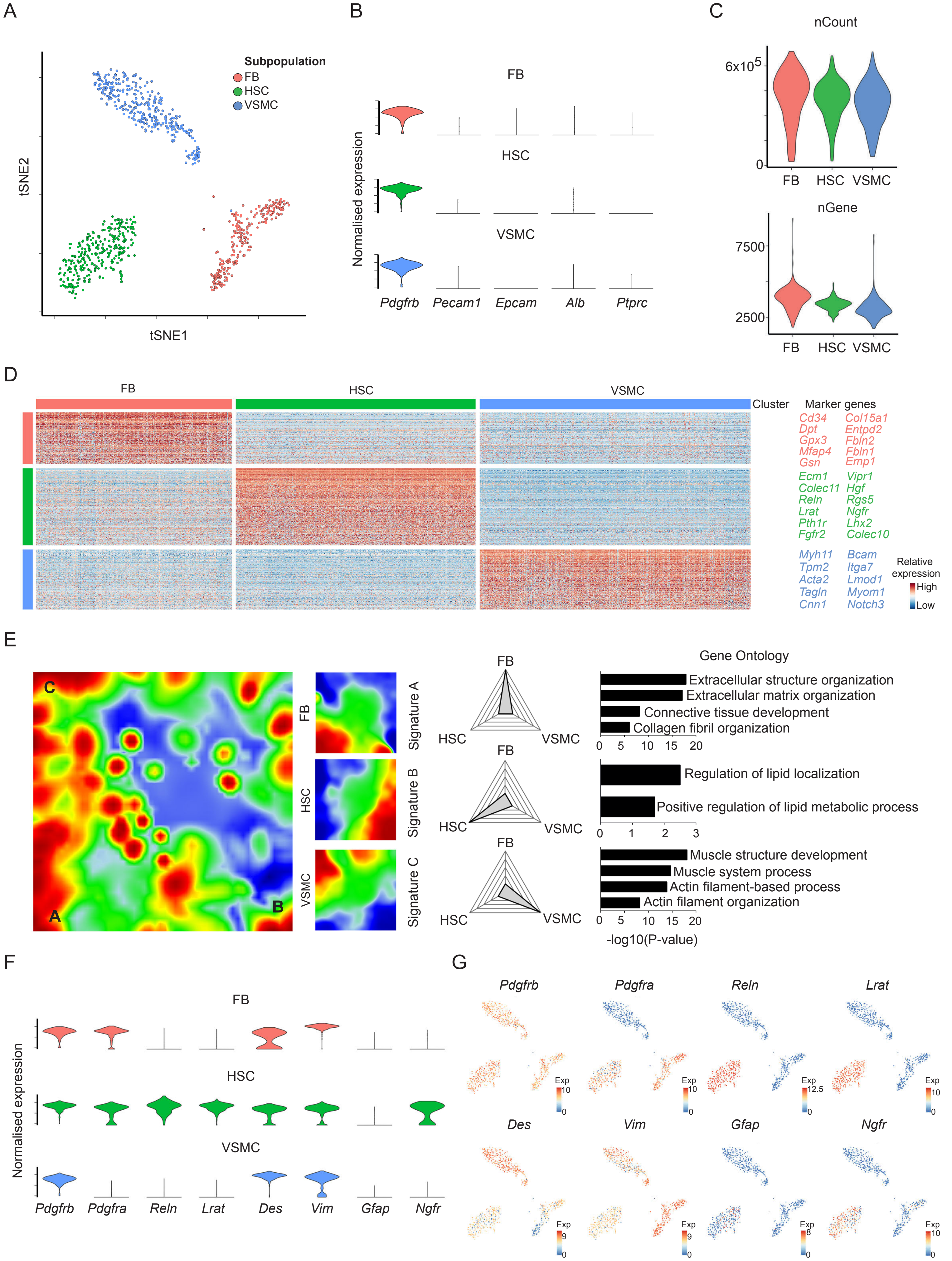

Supplemental Figure S3: SmartSeq2-based scRNA-seq of healthy mouse liver mesenchyme, related to Figure 1

(A) t-SNE visualisation: 905 mesenchymal cells (median nGene=3385, nCount=465,609) cluster into three subpopulations FB = fibroblasts, HSC = hepatic stellate cells, VSMC = vascular smooth muscle cells. (B) Violin plots: expression of mesenchymal and non-mesenchymal cell lineage markers in the healthy SSeq2 dataset (*Pecam1*: CD31; *Ptprc*: CD45). (C) Violin plots: number of total counts (nCount) and number of unique genes (nGene) expressed in mesenchymal cells in the healthy SSeq2 dataset. (D) Heatmap of relative expression: cluster marker genes (coloured by subpopulation), with exemplar genes labelled (right). Cells columns, genes rows. (E) Self-Organising Map (SOM; far left, 50x50 grid): smoothed scaled metagene expression. 19,612 genes, 2,500 metagenes, 23 signatures. A-C label metagene signatures overexpressed in one or more of the subpopulations (centre-left). Radar plots (centre-right): distribution of metagene signature A-C expression across the three subpopulations, with associated GO enrichment terms per signature (right). (F) Violin plots: traditional mesenchymal cell and HSC marker gene expression in the healthy SSeq2 dataset. (G) t-SNE visualisations: traditional mesenchymal cell and HSC marker gene expression in the healthy SSeq2 dataset.

Supplemental Figure S4

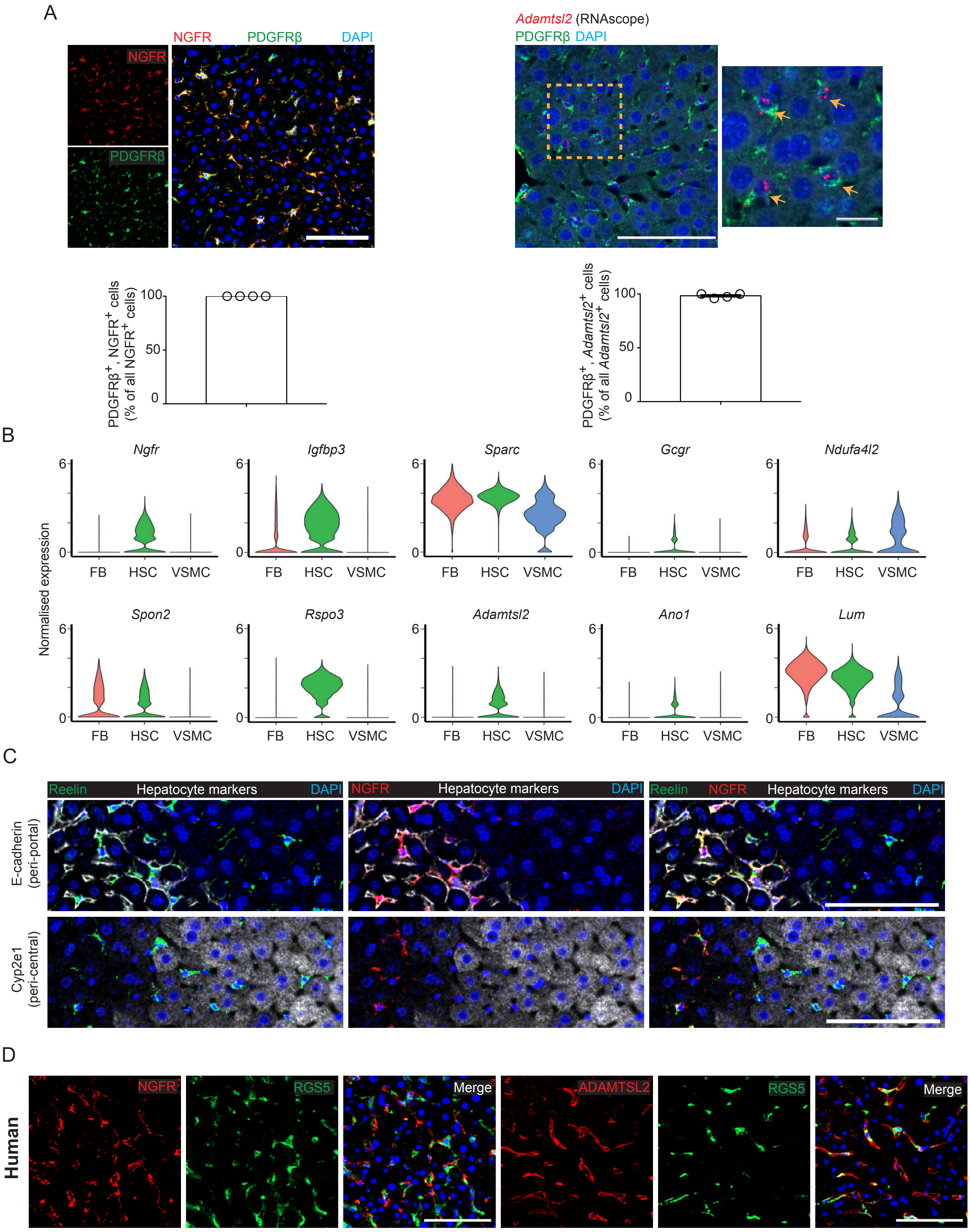

Supplemental Figure S4: Uncovering of HSC zonation across the healthy liver lobule, related to Figure 2

(A) Representative immunofluorescence and RNAscope images of healthy murine livers: NGFR / *Adamtsl2* (RNAscope) (red), PDGFRβ (green), DAPI (blue). Scale bar, 100µm. Yellow dashed line marks magnified area (scale bar, 20µm). Yellow arrows indicate *Adamtsl2*$^{+}$ (RNAscope) / PDGFRβ^{+} cells. Bar plots (below): NGFR / *Adamtsl2* (RNAscope) mesenchymal specificity (n=4); error bars SEM. (B) Violin plots: expression of top five genes with the highest gene weight loading on the zonation signature IC across the three subpopulations in uninjured liver. (C) Representative immunofluorescence images of healthy murine livers: Reelin (green), Hepatocyte markers – E-cadherin / Cyp2e1 (white), NGFR (red), DAPI (blue). Scale bar, 100µm. (D) Representative immunofluorescence images of healthy human livers: NGFR / ADAMTSL2 (red), RGS5 (green), DAPI (blue). Scale bar, 100µm.

Supplemental Figure S5

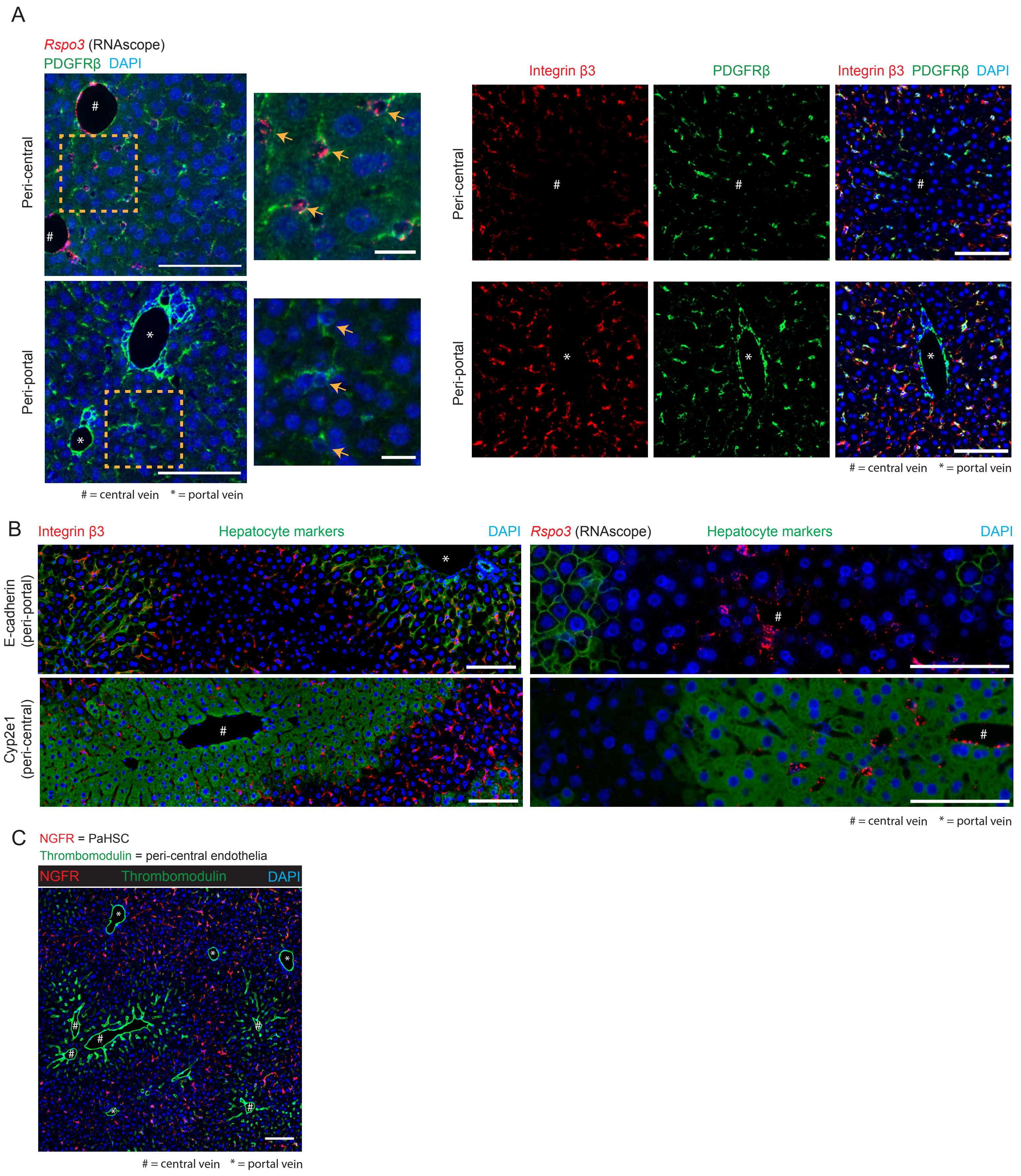

Supplemental Figure S5: Comparison of HSC and endothelial cells zonation, related to Figure 2

(A) Representative RNAscope and immunofluorescence images from peri-central and peri-portal regions of healthy murine livers: *Rspo3* (RNAscope) / Integrin β3 (red), PDGFRβ (green), DAPI (blue). Scale bar, 100μm; portal vein (*) and central vein (#) as indicated. Yellow dashed line marks magnified area (scale bar, 20μm). Yellow arrows indicate *Rspo3*$^{+}$ or *Rspo3*$^{-}$ (RNAscope) / PDGFRβ^{+} cells. (B) Representative immunofluorescence and RNAscope images of healthy murine livers: Integrin β3 / *Rspo3* (RNAscope) (red), Hepatocyte markers - E-cadherin / Cyp2e1 (green), DAPI (blue). Scale bar, 100μm; portal vein (*) and central vein (#) as indicated. (C) Representative immunofluorescence images of healthy murine livers: NGFR (red), Thrombomodulin (green), DAPI (blue). Scale bar, 100μm; central vein (#) and portal vein (*) as indicated.

Supplemental Figure S6

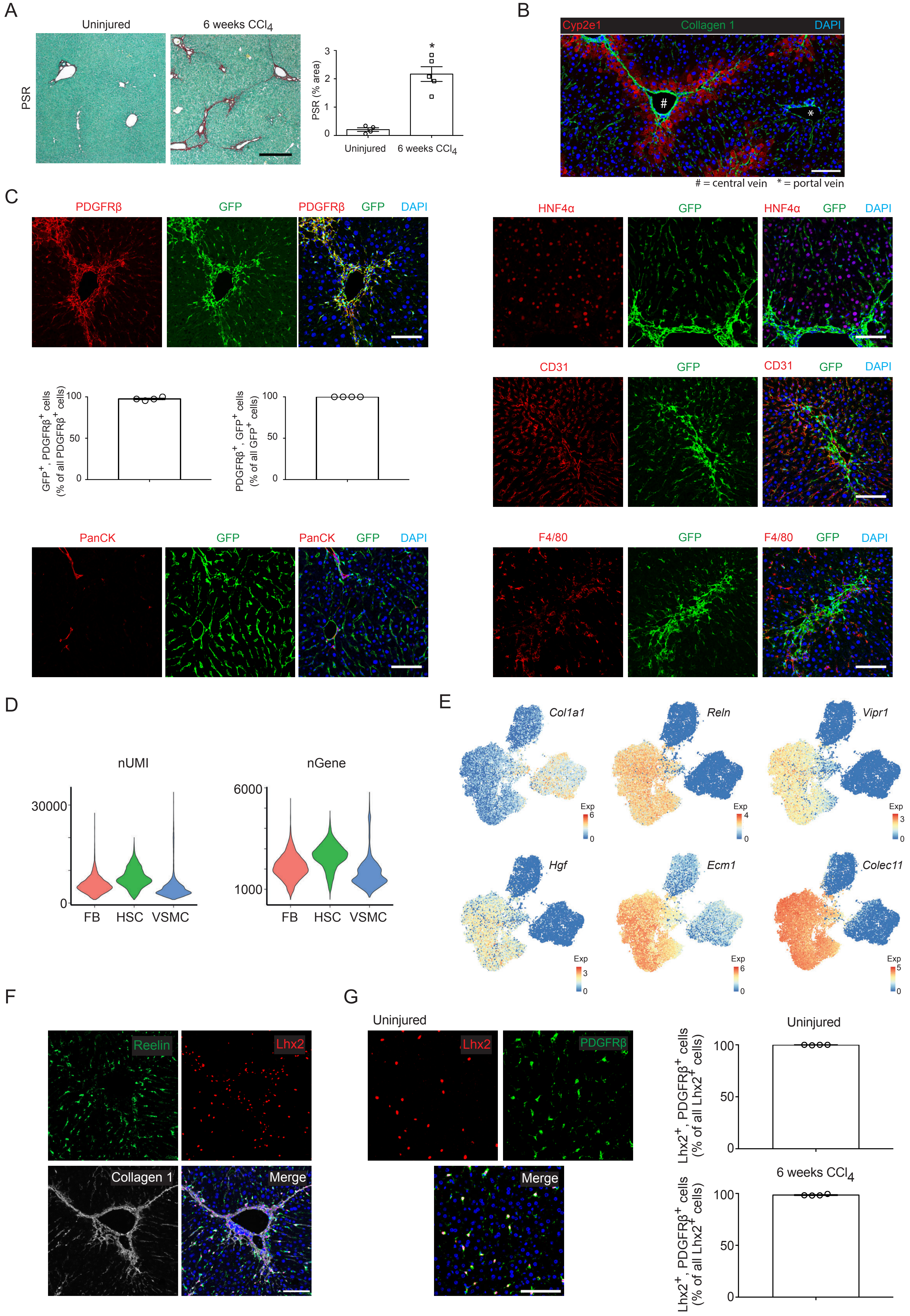

Supplemental Figure S6: Chronic CCl_4-induced fibrotic liver injury in *Pdgfrb*-BAC-eGFP mice, related to Figure 3

(A) Representative images and quantification of collagen deposition in fibrotic (6 weeks CCl_4) versus uninjured murine livers. Scale bar, 200µm. Bar plot (right): percentage picrosirius red staining (PSR) (uninjured n=4; 6 weeks CCl_4 n=5); error bars SEM, Mann-Whitney test, $*p < 0.05$. (B) Representative immunofluorescence images of fibrotic murine liver: Cyp2e1 (red), Collagen 1 (green), DAPI (blue). Scale bar, 100µm. (C) Representative immunofluorescence images of fibrotic murine livers: PDGFRβ / HNF4α / CD31 / PanCK / F4/80 (red), GFP (green), DAPI (blue). Scale bar, 100µm. Bar plot (centre): specificity and efficiency of *Pdgfrb*-BAC-eGFP reporting (n=4); error bars SEM. (D) Violin plots: number of total Unique Molecular Identifiers (nUMI) and number of unique genes (nGene) expressed in mesenchymal cells from healthy and fibrotic murine liver. (E) t-SNE visualisations: *Col1a1* and exemplar HSC marker genes expression in the combined healthy and fibrotic liver dataset. (F) Representative immunofluorescence images of fibrotic murine liver: Reelin (green), Lhx2 (red), Collagen 1 (white), DAPI (blue). Scale bar, 100µm. (G) Representative immunofluorescence images of uninjured murine liver: Lhx2 (red), PDGFRβ (green), DAPI (blue). Scale bar, 100µm. Bar plot (right): Lhx2 mesenchymal specificity in uninjured and fibrotic murine liver (n=4); error bars SEM.

Supplemental Figure S7

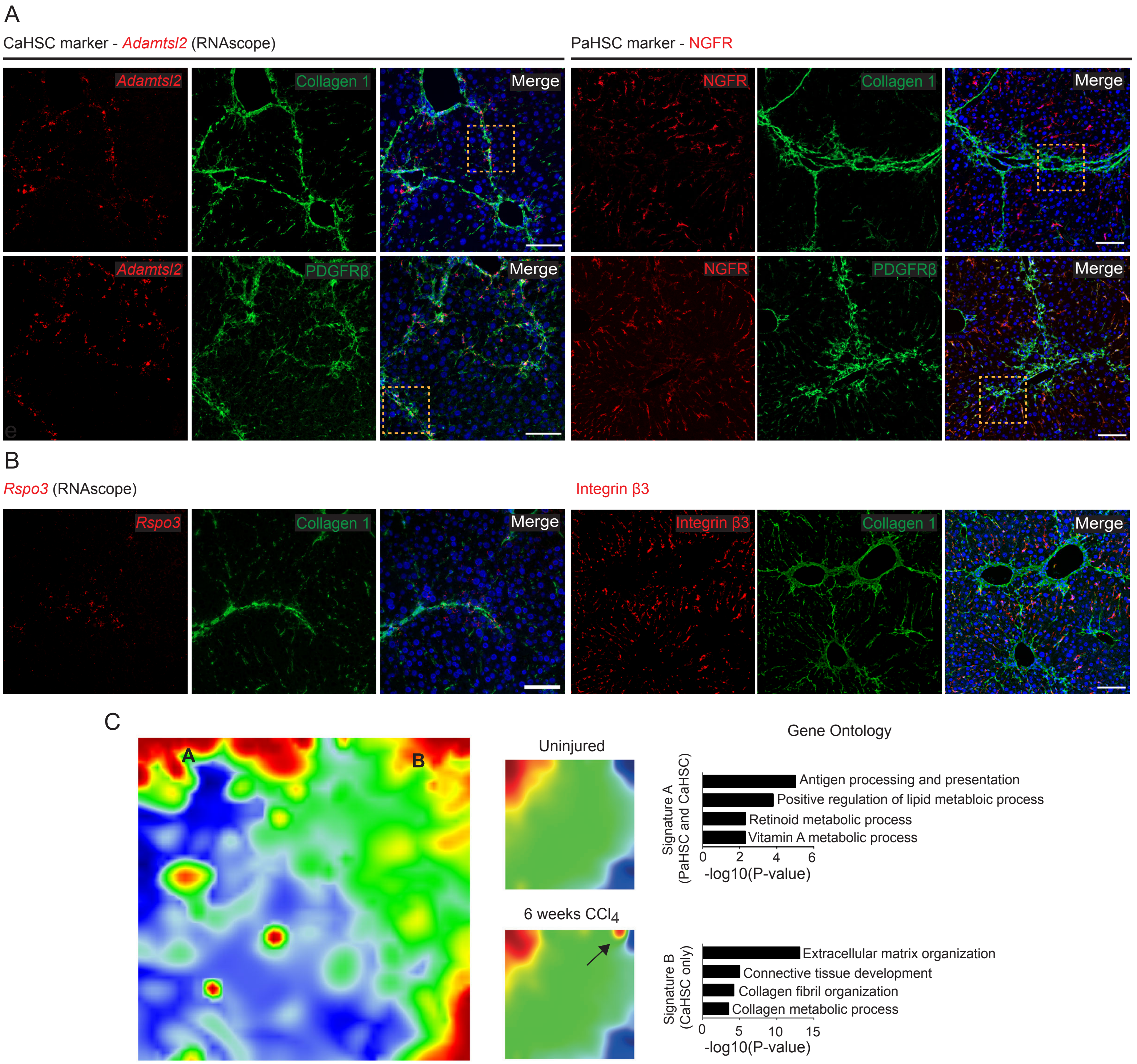

Supplemental Figure S7: CaHSC are the dominant pathogenic collagen-producing cells in a mouse model of fibrotic liver injury, related to Figure 4

(A) Representative immunofluorescence and RNAscope images of fibrotic (6 weeks CCl_4) murine livers: *Adamtsl2* (RNAscope) / NGFR (red), Collagen 1 / PDGFRβ (green), DAPI (blue). Scale bar, 100µm. Yellow dashed line marks magnified area displayed in Figure 4D. (B) Representative immunofluorescence and RNAscope images of fibrotic murine livers: *Rspo3* (RNAscope) / Integrin β3 (red), Collagen 1 (green), DAPI (blue). Scale bar, 100µm. (C) Self- Organising Map (SOM; far left, 60x60 grid) (left): smoothed scaled metagene expression in healthy and fibrotic murine mesenchyme. 17,159 genes, 3,600 metagenes, 14 signatures. A and B label metagene signatures of interest and are proportionally distributed among the HSC subpopulations (middle). Associated GO enrichment terms per signature (right).

Supplemental Figure S8

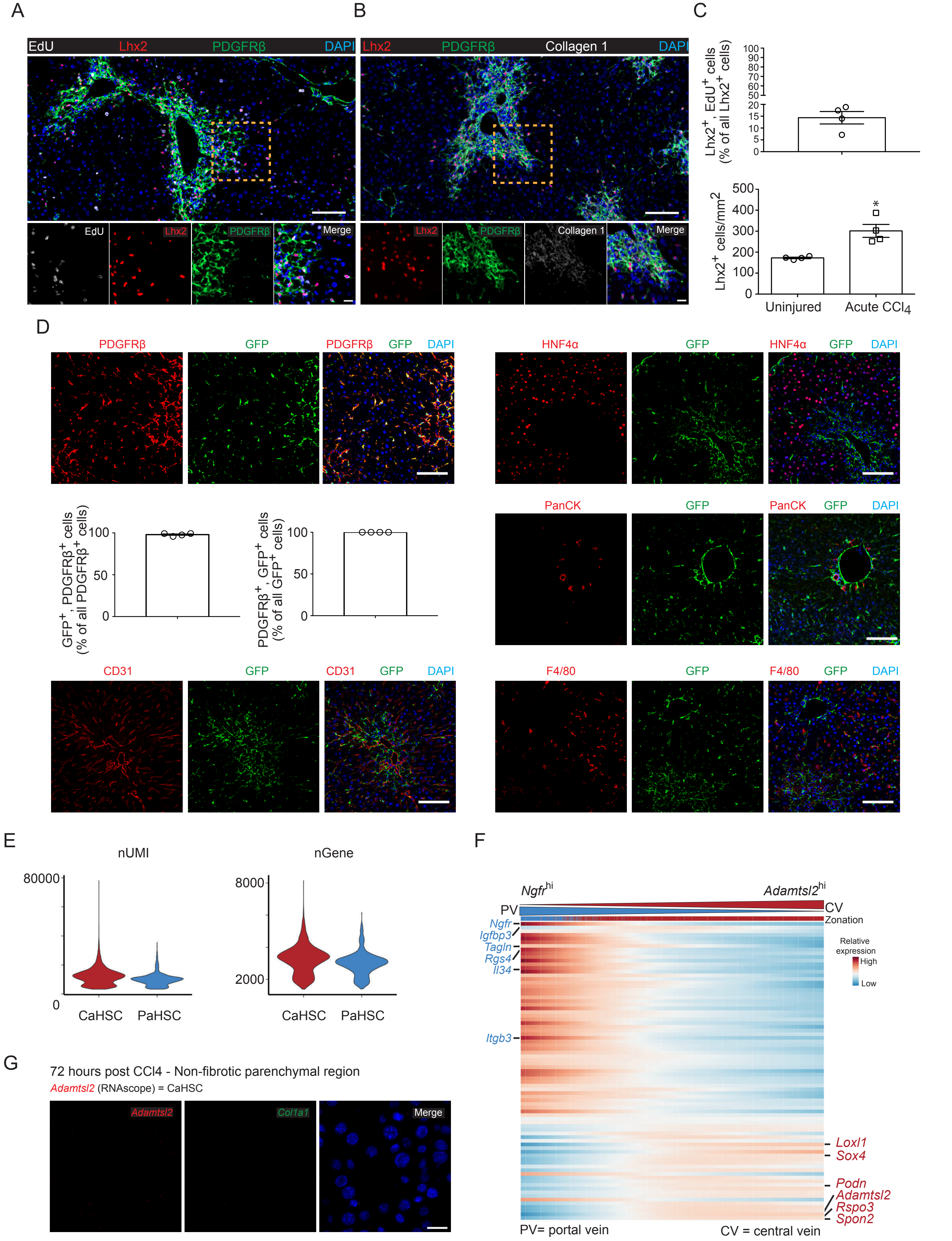

Supplemental Figure S8: Acute CCl_4-induced liver injury in *Pdgfrb*-BAC-eGFP mice, related to Figure 5

(A) Representative immunofluorescence images of acute CCl_4-induced injury (72 hours following single CCl_4 injection) murine liver: EdU (white), Lhx2 (red), PDGFRβ (green), DAPI (blue). Scale bar, 100µm. Yellow dashed line marks magnified area (scale bar, 20µm). (B) Representative immunofluorescence images of $Lhx2^+$ HSCs in acute CCl_4-induced injury versus uninjured murine liver: Lhx2 (red), PDGFRβ (green), Collagen 1 (white) DAPI (blue). Scale bar, 100µm. Yellow dashed line marks magnified area (scale bar, 20µm). (C) Bar plot (top): percentage EdU^+ $Lhx2^+$ cells (n=4); error bars SEM. Bar plot (below): number of $Lhx2^+$ cells per mm^2 in uninjured versus CCl_4 treated (acute) liver (uninjured n=4; acute CCl_4 n=4); error bars SEM, Mann-Whitney test, $*p < 0.05$. (D) Representative immunofluorescence images of liver following acute CCl_4-induced injury: PDGFRβ / HNF4α / PanCK / CD31 / F4/80 (red), GFP (green), DAPI (blue). Scale bar, 100µm. Bar plots (centre): specificity and efficiency of *Pdgfrb*-BAC-eGFP reporting (n=4); error bars SEM. (E) Violin plots: number of total Unique Molecular Identifiers (nUMI) and number of unique genes (nGene) expressed in CaHSC and PaHSC following acute CCl_4-induced liver injury. (F) Heatmap of relative expression: cubic smoothing spline curves fitted to previously-defined markers of zonation in murine HSC, ordered by expression of *Ngfr*-associated (portal vein-associated) signature and annotated by cell condition. Cells columns, genes rows. (G) Magnified RNAscope image of a parenchymal region of murine liver following acute CCl_4-induced liver injury: *Adamtsl2* (RNAscope; red), *Col1a1* (RNAscope; green), DAPI (blue). Scale bar, 20µm.

Supplemental Figure S9

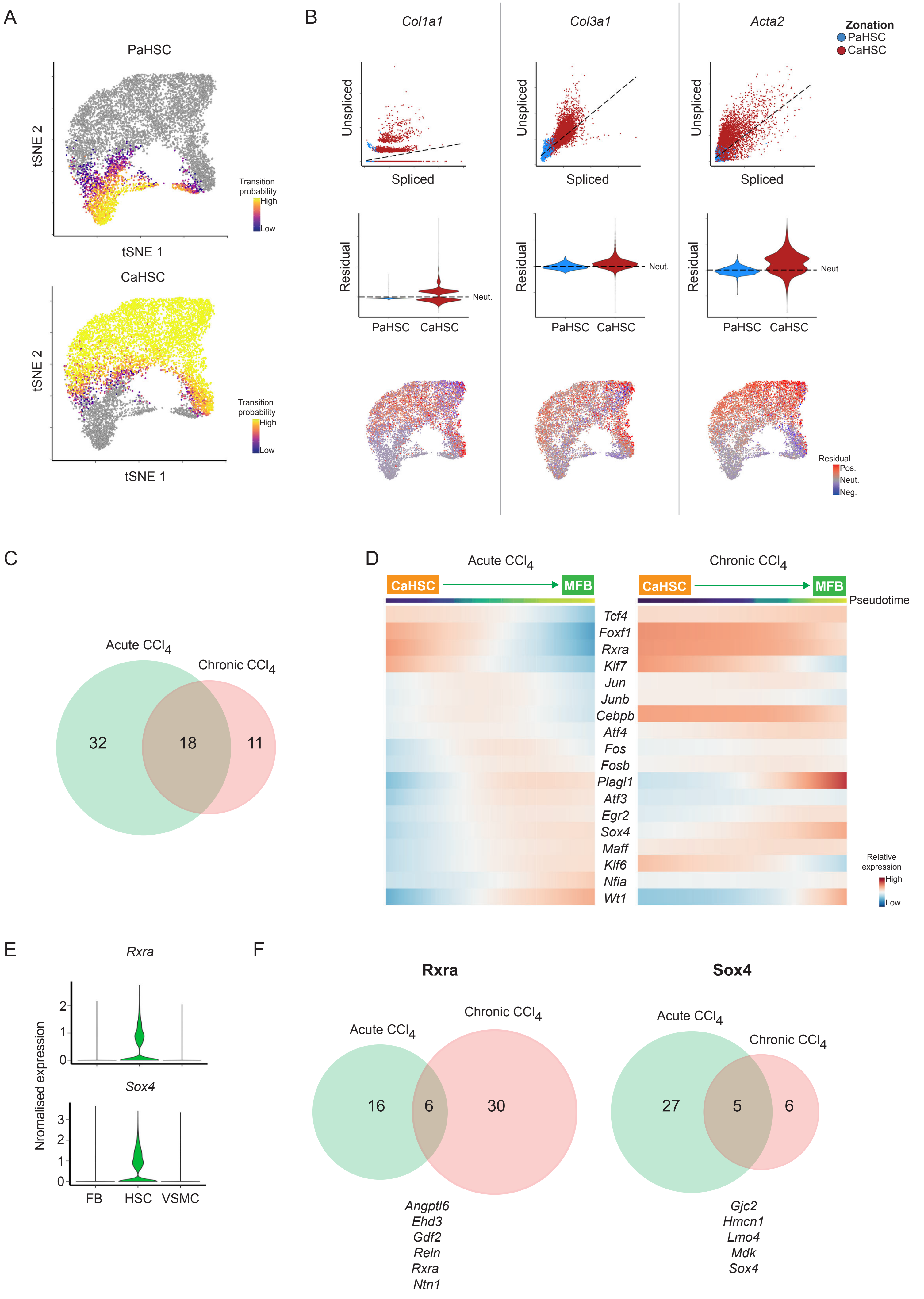

Supplemental Figure S9: Pseudotemporal dynamics and transcriptional regulation in HSC, related to Figure 6

(A) t-SNE visualisations: transition probabilities per HSC cluster, indicating for each cell the likelihood of transition into either PaHSC (top) or CaHSC (bottom), calculated using RNA velocity (yellow high; purple low; grey below mean threshold). (B) Unspliced-spliced phase portraits (top row), cells coloured by PaHSC or CaHSC for pro-fibrogenic genes *Col1a1*, *Col3a1*, and *Acta2*. Cells plotted above or below the steady-state (black dashed line) indicate increasing or decreasing expression of gene, respectively. Unspliced residuals (centre and bottom rows), positive (above dashed line; red) indicating expected upregulation, negative (below dashed line; blue) indicating expected downregulation for genes. (C) Venn diagram: number of regulons identified by SCENIC as differentially expressed along acute (72 hours post single CCl_4 injection) and chronic (6 weeks CCl_4) CCl_4-induced liver injury (green=acute, red=chronic). (D) Heatmaps of relative expression: cubic smoothing spline curves fitted to transcription factors differentially expressed and conserved across transition from quiescent CaHSC to myofibroblast following acute (left) or chronic (right) CCl_4-induced liver injury. (E) Violin plots: expression of transcription factors *Rxra* and *Sox4* across mesenchymal populations in chronic CCl_4-induced liver injury. (F) Venn diagrams: number of regulatory target genes identified by SCENIC as co-expressed with transcription factors *Rxra* (left) or *Sox4* (right) (green=acute, red=chronic). List of shared target genes displayed below.

Supplemental Figure S10

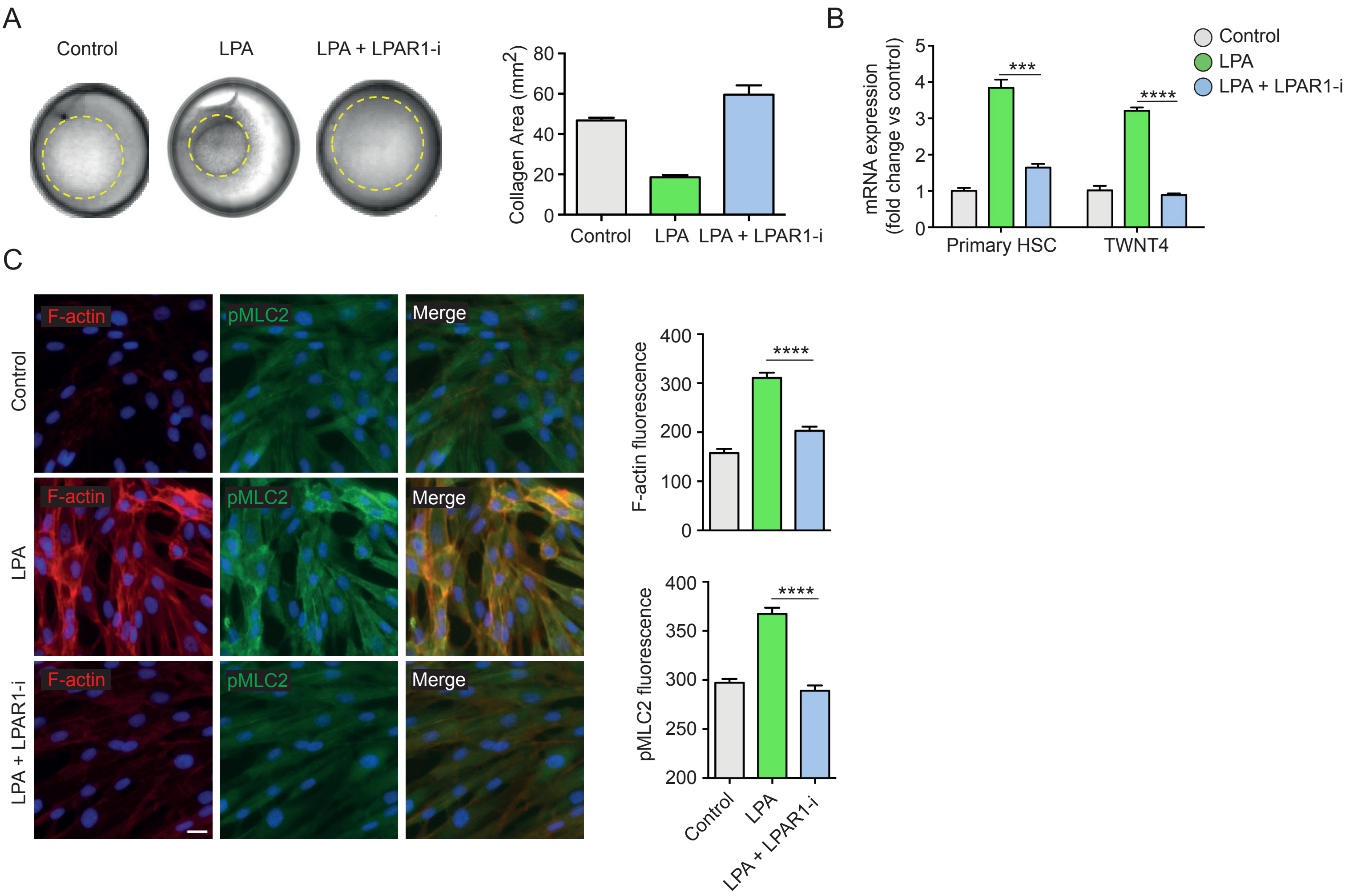

Supplemental Figure S10: LPAR1 antagonism inhibits LPA-induced human HSC contractility and activation, related to Figure 7

(A) Collagen contraction assay: representative images of TWNT4 cells seeded in a collagen matrix and treated with 50μM LPA ± LPAR1 antagonist (LPAR1-i; 3μM). Bar plot (right): Mean collagen area (n=2) per treatment group; error bars SEM. (B) Connective tissue growth factor (CTGF) mRNA expression in primary human HSC (n=3 per treatment group) and TWNT4 cells (n=3 per treatment group) treated with 10μM LPA ± LPAR1 antagonist (LPAR1-i; 3μM); error bars SEM, Unpaired ttest, ***p < 0.001, ****p < 0.0001. (C) Representative immunofluorescence images of TWNT4 cells treated with 10μM LPA ± LPAR1 antagonist (LPAR1-i; 1μM) pre-treatment: F-actin (red), pMLC2 (green), Hoechst 33342 (blue). Scale bar, 20μm. Bar plots (right): mean fluorescence (a.u) of F-actin or pMLC2 (n=9) per treatment group; error bars SEM, Mann-Whitney test, ****p < 0.0001.
